# Carbon dioxide dynamics in a lake and a reservoir on a tropical island (Bali, Indonesia)

**DOI:** 10.1371/journal.pone.0198678

**Published:** 2018-06-11

**Authors:** Paul A. Macklin, I. Gusti Ngurah Agung Suryaputra, Damien T. Maher, Isaac R. Santos

**Affiliations:** 1 National Marine Science Centre, Southern Cross University, Coffs Harbour, New South Wales, Australia; 2 School of Environment, Science and Engineering, Southern Cross University, Lismore, New South Wales, Australia; 3 Department of Analytical Chemistry, Universitas Pendidikan Ganesha, Singaraja, Bali, Indonesia; Beijing Normal University, CHINA

## Abstract

Water-to-air carbon dioxide fluxes from tropical lakes and reservoirs (artificial lakes) may be an important but understudied component of global carbon fluxes. Here, we investigate the seasonal dissolved carbon dioxide (CO_2_) dynamics in a lake and a reservoir on a tropical volcanic island (Bali, Indonesia). Observations were performed over four seasonal surveys in Bali’s largest natural lake (Lake Batur) and largest reservoir (Palasari Reservoir). Average CO_2_ partial pressures in the natural lake and reservoir were 263.7±12.2 μatm and 785.0±283.6 μatm respectively, with the highest area-weighted partial pressures in the wet season for both systems. The strong correlations between seasonal mean values of dissolved oxygen (DO) and *p*CO_2_ in the natural lake (r^2^ = 0.92) suggest that surface water metabolism was an important driver of CO_2_ dynamics in this deep system. Radon (^222^Rn, a natural groundwater discharge tracer) explained up to 77% of the variability in *p*CO_2_ in the shallow reservoir, suggesting that groundwater seepage was the major CO_2_ driver in the reservoir. Overall, the natural lake was a sink of atmospheric CO_2_ (average fluxes of -2.8 mmol m^-2^ d^-1^) while the reservoir was a source of CO_2_ to the atmosphere (average fluxes of 7.3 mmol m^-2^ d^-1^). Reservoirs are replacing river valleys and terrestrial ecosystems, particularly throughout developing tropical regions. While the net effect of this conversion on atmospheric CO_2_ fluxes remains to be resolved, we speculate that reservoir construction will partially offset the CO_2_ sink provided by deep, volcanic, natural lakes and terrestrial environments.

## Introduction

Lakes and reservoirs cover 2.2% of the global surface area [1]. Although relatively small in aerial extent, lakes play a significant and increasingly important role in the global carbon cycle [2, 3]. Estimates of the global net CO_2_ flux for lakes and reservoirs is ~0.3 Pg C yr^-1^ (range 0.06 to 0.84 Pg C yr^-1^) but there are uncertainties in the number and area of small lakes [1]. With climate change, it is likely that there will be a global changes in lake abundance. It is predicted that there will be losses of natural lakes in regions where the climate is becoming drier [4] and increases in reservoir construction [5] in regions with rapidly expanding populations such as Southeast Asia [4]

Lakes and reservoirs modify freshwater flows of inland waters and alter CO_2_ fluxes by retaining ~50% of the global carbon transported to the oceans [4]. Estimates of global reservoir numbers have varied significantly from 25, 410 to 515,149 [6, 7], the latter study including smaller reservoirs. In spite of this variability, reservoirs are important compared to natural lakes (n = 304 million) with an estimated 277 million small lakes (0.001–0.01 km^2^) [7]. Millions of smaller reservoirs (<0.5 km^2^; [8]) are not accounted for in global carbon budgets, and reservoir construction is increasing [8] resulting in more terrestrial carbon entering and becoming trapped in lakes and reservoirs, and changes in landscape CO_2_ emissions [9]. These shifts in CO_2_ emissions are further compounded by predicted increases in extreme weather events such as flood and droughts [10] as well as watershed degradation and weathering [11, 12].

Global assessments of CO_2_ fluxes from lakes and reservoirs are not evenly distributed with temperate and boreal zones such as Europe and Northern America largely over-represented [13]. Tropical systems [14] and the southern hemisphere [15] are under-represented. Tropical systems comprise ~40% of the global surface area of reservoirs. However, ~70% of CO_2_ fluxes from reservoirs are thought to originate in tropical regions [6]. As a result, tropical reservoirs are recognised as disproportionally large sources of CO_2_ to the atmosphere [6, 16]. Compared to temperate lakes, higher water temperatures in tropical regions result in higher organic matter decomposition rates, resulting in higher CO_2_ production and emissions than their temperate counterparts [4, 6].

CO_2_ dynamics in both lakes and reservoirs are often driven by a combination of internal processes such as photosynthesis and respiration, as well as allochthonous inputs [17]. Allochthonous sources of CO_2_ include weathering, soil organic matter and terrestrial root respiration [18, 19] and precipitation of carbonate or silicate minerals [20]. Surface water runoff [21, 22] and groundwater discharge [19, 23–25] can directly deliver terrestrial organic matter to aquatic systems, which is subsequently stored in lake sediments, exported downstream, remineralised or released to the atmosphere [4]. Since groundwater is often highly supersaturated in CO_2_ when compared to surface waters and the atmosphere [26], several recent studies identified groundwater seepage as a major conduit of CO_2_ to lakes [27–29].

Here, we contribute to filling knowledge gaps of inland water CO_2_ dynamics in tropical regions, where there are fewer data on CO_2_ outgassing rates and factors controlling this efflux than the more comprehensively studied temperate regions. We measured *p*CO_2_ and estimate fluxes at the water-air interface along with potential drivers in a natural lake and a reservoir in Bali, Indonesia, one of the world’s fastest growing tourist economies. Although Indonesia has been identified as having a high potential for groundwater recharge [30], comprehensive studies including groundwater-derived CO_2_ seepage in Indonesian lakes have not been conducted to date. We hypothesize that groundwater seepage may release CO_2_ to surface waters and that CO_2_ concentrations will be higher in the wet season due to a relative increase in groundwater flow. Seasonal surveys of radon (a natural groundwater discharge tracer) and CO_2_ are used to test this hypothesis.

## 2. Material and methods

### 2.1 Area description

**Fig 1:**
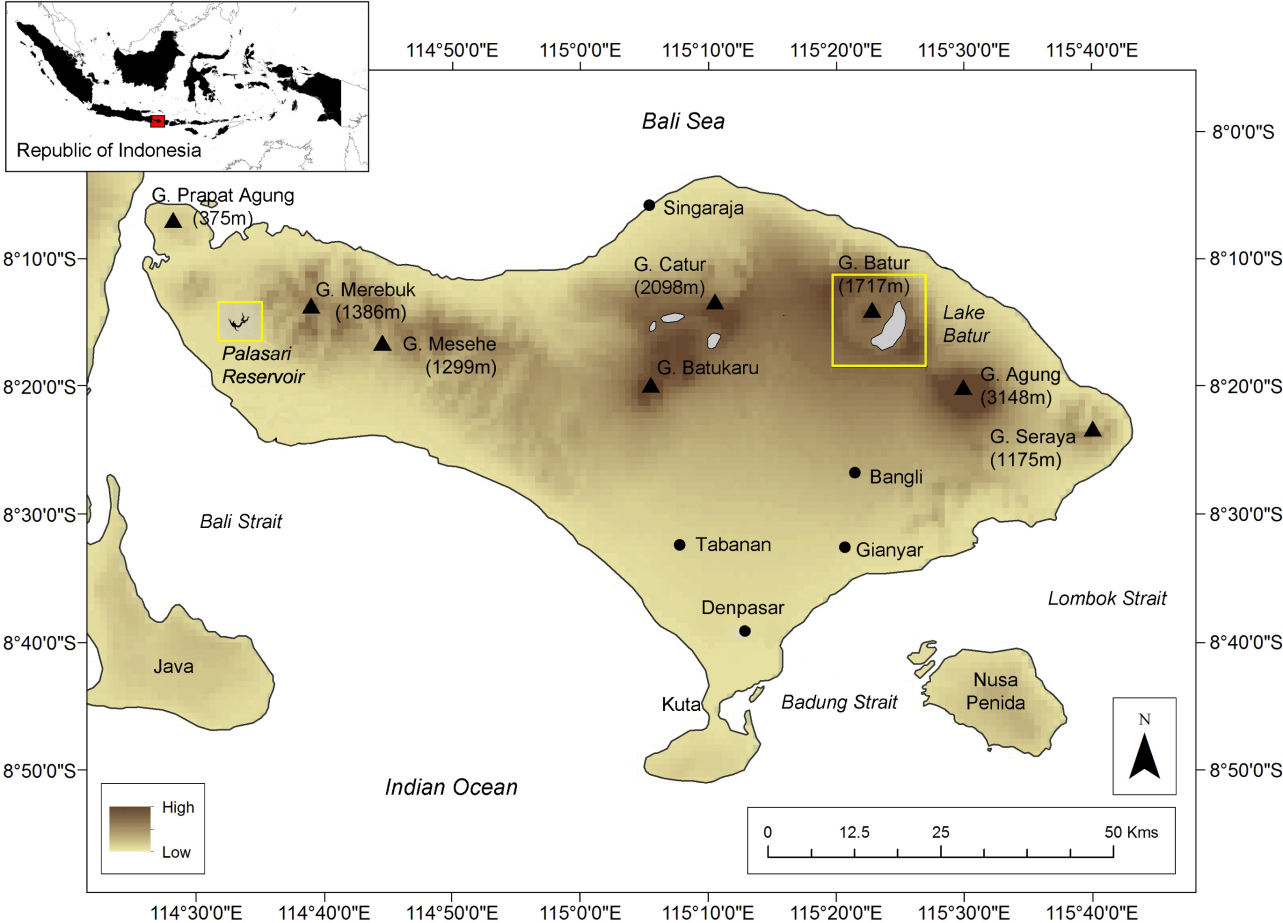
Palasari Reservoir (left) and Lake Batur (right) study sites (highlighted by yellow boxes) on Bali Island, Indonesia.

Indonesia has 521 natural lakes and over 100 reservoirs which cover ~ 21,000 km^2^ [31]. Bali Province is bounded by the Java Sea, the Lombok Strait, the Indian Ocean and the Bali Strait. It has 8 groundwater basins, 1273 springs, 4 lakes, 4 reservoirs, 5 ponds, and ~162 rivers (www.blh.baliprov.go.id/). Utilisation of rivers as a water source is widely unviable as the flow is intermittent, with <11% of the rivers flowing in the dry season (IDEP, 2009). Historically, Bali experiences a dry season from May to September, a transition season in October, wet season from October to April followed another transition season in March. This paper focuses on two systems on the island of Bali: Palasari Reservoir (area = 10, 056 m^2^; mean depth = 16.4 m) and Lake Batur (area = 17, 180, 000 m^2^; mean depth = 50.8 m) (Fig 1 and Table 1). To our knowledge, these are the first observations of CO_2_ in Bali’s lakes and reservoirs.

**Table 1:** Morphometric and hydrological characteristics of Lake Batur and Palasari Reservoir.

Site	Units	Lake Batur	Palasari Reservoir
Latitude		8.25888°S	8.25310°S
Longitude		115.40825°E	114.54920°E
Fieldtrip dates	Drought	22/11/2015	12/10/2015
	Dry	06/01/2016	07/01/2016
	Wet transition	14/03/2016	18/03/2016
	Wet	24/06/2016	29/06/2016
Regional soils	** **	Grey Regosol	Brown Latisol and Litosol
Lake level	m a.s.l.	1017	90
Watershed area (Wa)	m^2^	105,350,000	4,230,000
Lake area (La)	m^2^	17,180,000	10,056
Watershed area-lake area ratio		6.13	420.64
Max. depth	m	88	14–19 (Range in depths between surveys)
Mean depth	m	50.8	16.4
Water volume	m^3^	815,380,000	8,000,000 (max)
Rainfall (sampling year)	mm/yr^-1^	1637.0	1716.6
Rainfall (historical average)	mm	2703.6	1731.1
Lake perimeter	km	21.4	10.7
Mixing type		Monomictic	Meromictic Polymictic
Location permeability	cm/sec	NA	10^−2^–10^−5^

Lake Batur is Bali’s largest and deepest natural lake (Fig 1). It is a confined active caldera lake formed in the depression of the collapsed volcano walls of Mt Batur with a small watershed/lake area ratio (~6:1). Topography is undulating lowlands to Mt Batur (north), and steep hills and crater walls (north, east and south). The geology is comprised of old Buyan-Bratan and Batur volcanics with basalt to basaltic andesite lavas and pyroclastic deposits underlying, and inter layered Batur Ignimbrite, (permeable when fractured with a secondary opening) and Grey Regosol soils which are vulnerable to soil erosion. The walls of the lake drop steeply to a maximum depth of 88 m, with a narrow littoral zone (Table 1) and diurnal microstratifactions and thermal and chemostratifications. Inflows to the lake includes Batur Spring, deep groundwater springs in the pyroclastic flow slope and rainfall. Small scale settlements, agriculture, aquaculture, geothermal springs and the Mt. Batur pyroclastic flow slope are found on the west side of the lake.

Palasari Reservoir is Bali’s largest reservoir, located on the west coast of Bali, ~6 km downstream of Mt Sangiang (1,004 m a.s.l.). The geology of the region is dominated by the quaternary Palasari Formation which includes Palasari conglomerate, sandstone, calcareous sandstone and limestone reef. The topography is low-lying hills with regional soils dominated by Brown Latisol (http://ppsp.nawasis.info/) which are highly permeable with vegetation cover. Without vegetation cover the soil is vulnerable to erosion and rapidly becomes impermeable. The Palasari Reservoir has a large watershed area-lake area ratio (~420:1) with surrounding land use dominated by small scale agriculture. Upstream of the reservoir is protected forest with a short (<5 km) topographic transition from hilly to mountainous terrain. The reservoir is a 27 year old, rock fill type dam with a central clay land fill core of 40 m. It functions as flood control and supplies irrigation water for ~13 km^2^ of rice fields downstream. Although there may be receiving inflow from the Sangiang Gode and Palareja Rivers, during the sampling period there was no notable surface water inflow into and out of the system (Table 1).

### 2.2 Approach and methods

**Fig 2:**
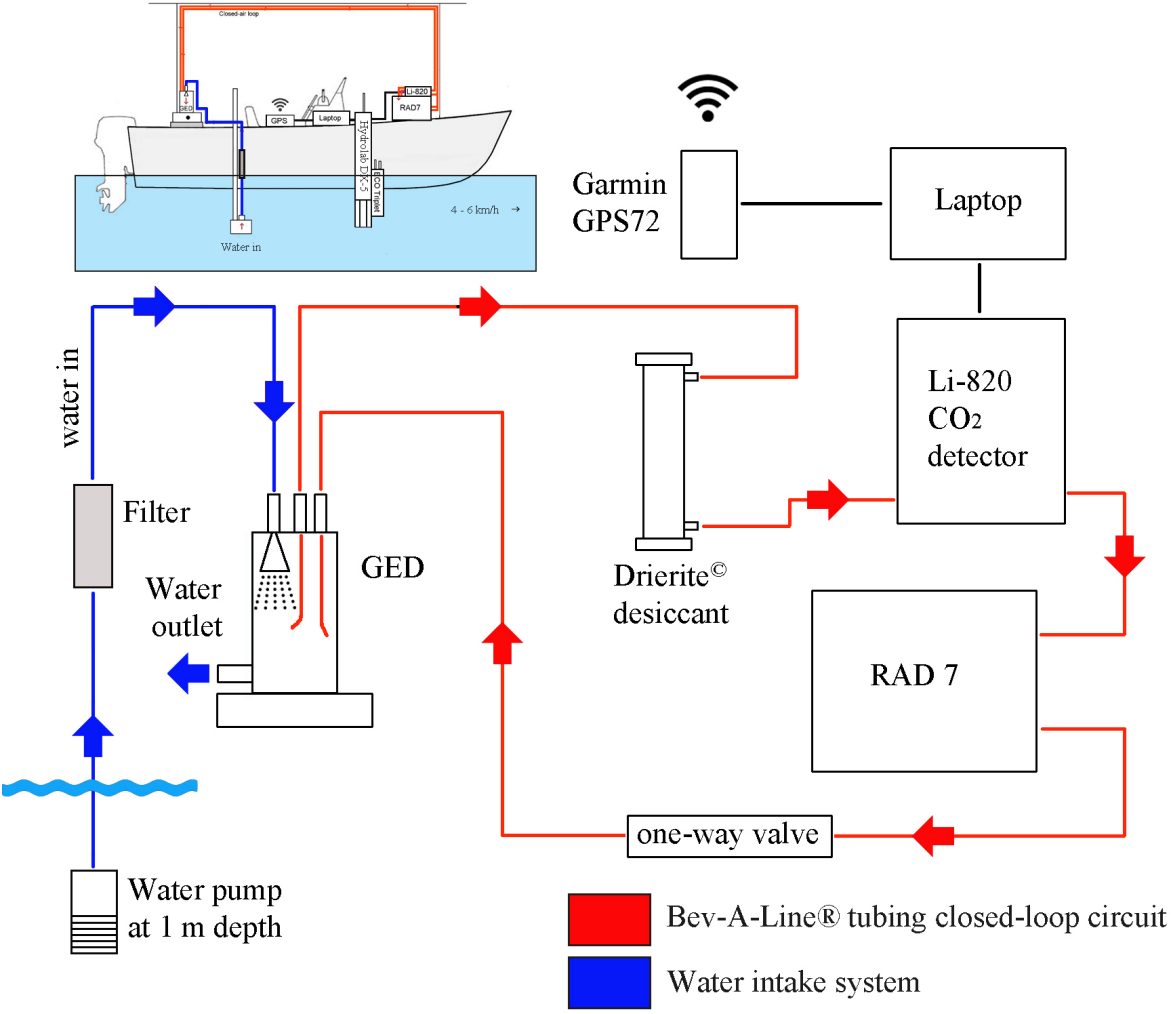
Coupled radon and carbon dioxide system schematic layout [26].

We performed 4 seasonal surveys in Lake Batur and Palasari Reservoir using automated instrumentation (Fig 2). Instrumentation was installed on a small research vessel driven at 4–6 km/h to produce high spatial resolution sampling. The vessel was stopped or slowed down at sites of high interest such as areas where the landscape was modified, near stream inlets, around visible changes in nearshore vegetation, and large transitions in water depth. Location was logged continuously by a Garmin GPS72 or Maverick Pro 2.61 Android GPS.

Water column *p*CO_2_ and ^222^Rn concentrations were measured from a depth of ~1 meter using a portable Li-820 CO_2_ detector (calibrated with 0, 400 and 10 000 ppm standards) and a radon-in-air monitor calibrated prior to deployment by the manufacturer (RAD7, Durridge; Fig 2). The detectors were connected with a closed-air-loop and an equilibrator spray chamber [26, 32] with the air stream dried with a desiccant column of Drierite. Water was continually pumped from a submersible bilge pump at about 3 L/min^-1^ into a shower head gas equilibration device (GED). A closed-air-loop was created between the GED and gas detectors which measure the gas concentrations in the air stream. Air was pumped into the RAD7 radon detector at 1 L min^-1^ by the RAD7 internal pump. The dissolved gas concentrations were determined by the gas solubility and temperature [33, 34].

Measurements of temperature, conductivity and dissolved oxygen were undertaken using a Hydrolab DS-5 multiparameter water quality sonde, calibrated prior to each deployment at 1 min intervals to measure pH (± 0.02 units), salinity (± 0.02 ppt), dissolved oxygen (± 0.2 mg L-1), and water temperature (± 0.10°C). pH was calibrated with 4, 7 and 10 buffer solutions (NBS scale) while conductivity was calibrated with deionised water and a 1413 μS cm^-1^ standard. Chlorophyll a was measured at 1 minute intervals with a WETlabs Eco triplet fluorometer equipped with a copper brush wiper to prevent biofouling of sensors and calibrated by the manufacturer using quinine dehydrate. Meteorogical data was sourced from Denpasar Ngurah Rai Weather Station 972300 (S08.749; E115.167).

The CO_2_ flux across the water–air interface was calculated according to Wanninkhof (1992) [35]:

$$F_{CO2}=k*K_{H}*\Delta pCO_{2},$$ (1)

where k is the CO_2_ gas transfer velocity, K_H_ is the solubility of CO_2_ [33] and ΔpCO_2_ is the difference between the partial pressure of *p*CO_2_ in water and air. To calculate k, we used the average of six parameterizations to provide a reasonable range in evasion rate estimates (Table 2). Positive values represent a water-to-air CO_2_ flux and negative values represent an air-to-water flux. Water-to-air CO_2_ fluxes were calculated by using five minute sampling times for *p*CO_2_ and average annual windspeeds to reduce wind bias for the natural lake and reservoir, respectively. Integrated aereal CO_2_ fluxes were calculated using the Spline-with-Barriers method [36] to prevent bias related to different research vessel speeds and time spent stationary.

**Table 2:** Six wind-speed based parameterization formulas with respective authors, where k is the transfer velocity (cm h^−1^), u is the wind speed (ms^−1^) at a height of 10 m and Sc is the Schmidt number of CO_2_ at in situ temperature and salinity.

Authors		Formula	Code	Ecosystem
Wanninkhof (1992) [38]	(1)	k = 0.31u_10_^2^ (Sc/660)^‐0.5^	W92	Lake
MacIntyre et al. (1995) [57]	(2)	k = 0.45u_10_^1.6^ (Sc/600)^-0.5^	M95	Lake
Cole & Carico (1998) [58]	(3)	k = 2.07 + 0.215u_10_^1.7^ (Sc/600)^-0.5^	C&C98	Lake
McGillis et al. (2001) [59]	(4)	k = 3.3 + 0.026u_10_^3^ (Sc/600)^-0.5^	M01	Lake
Crusius and Wanninkhof (2003) [60]	(5)	k = 0.168 + 0.228u_10_^2.2^ (Sc/600)^-0.5^	C&W03	Lake
Cole et al. (2010) [61]	(6)	k = 0.497 + 0.0064u_10_^1.8^ (Sc/600)^-0.5^	C10	Lake

Permits and permissions for Palasari Reservoir and Lake Batur were provided by the Indonesian Foreign Research Permit Secretariat, Ministry of Research, Technology and Higher Education of the Republic of Indonesia (RISTEKDIKTI), the Directorate General of Water Resources, the Indonesian Ministry of Public Works (DGWRD) and the Governor of Bali, I Made Mangku Pastika. Field studies did not involve endangered or protected species.

## 3. Results

**Fig 3:**
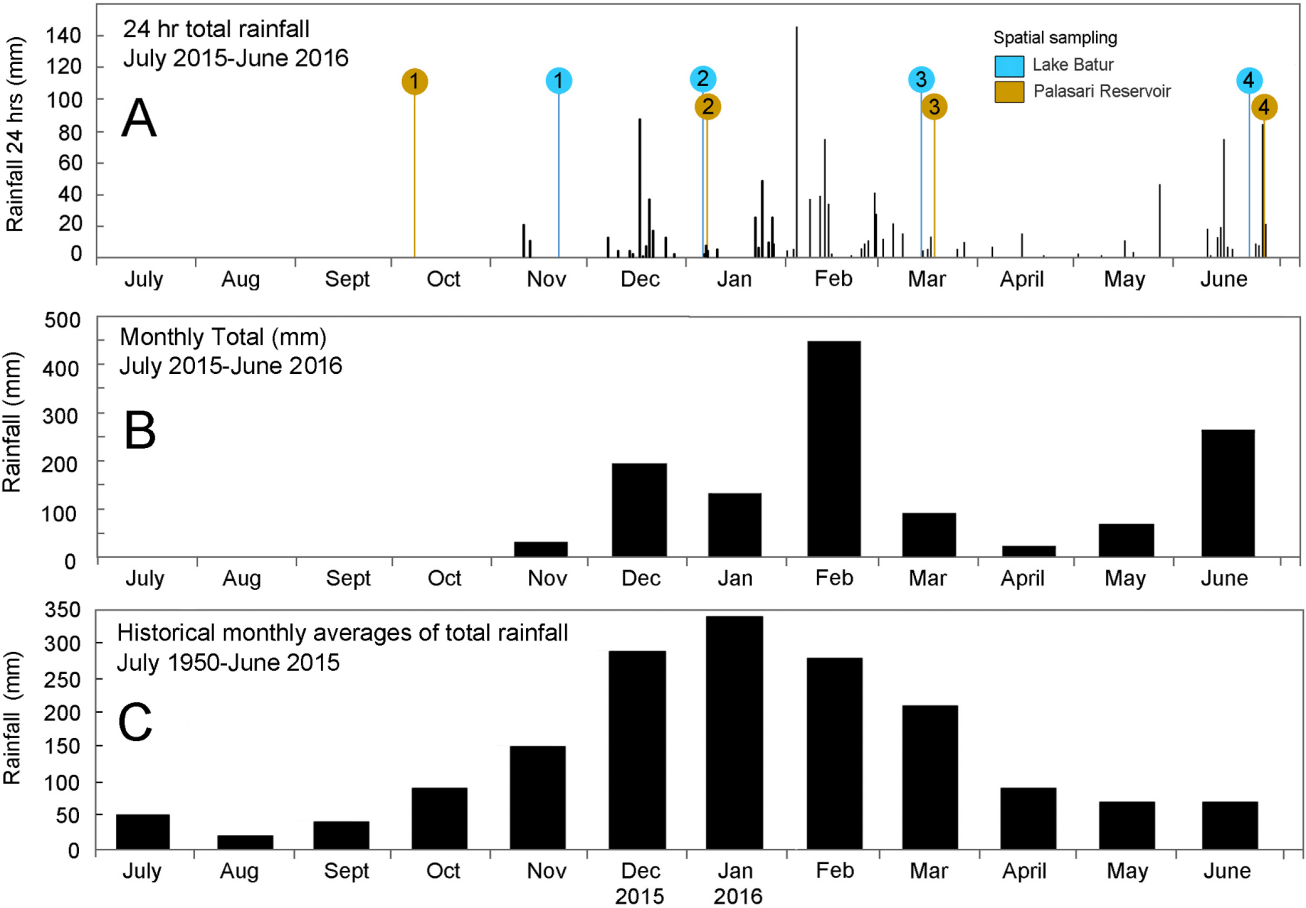
Rainfall time series compiled from raw rainfall datasets, indicating (A) underway seasonal sampling timeline for the natural lake, Lake Batur (blue) and the reservoir, Palasari Reservoir (brown); (B) rainfall monthly total from July 2015 to June 2016 and (C) historical monthly rainfall averages from 1950 to 2015 for Denpasar, Bali. Data accessed from NOAA, National Centre for Environmental Information (www.ncdc.noaa.gov/).

Both study sites experienced an extended drought period during the 2015 dry season (Fig 3) with no rainfall recorded 3 months prior to initial sampling in November 2015. In contrast the 2016 dry season (May-September) recorded significantly more rainfall than historical averages. This created a sampling period with initial dry conditions transitioning to wetter conditions in both systems.

**Fig 4:**
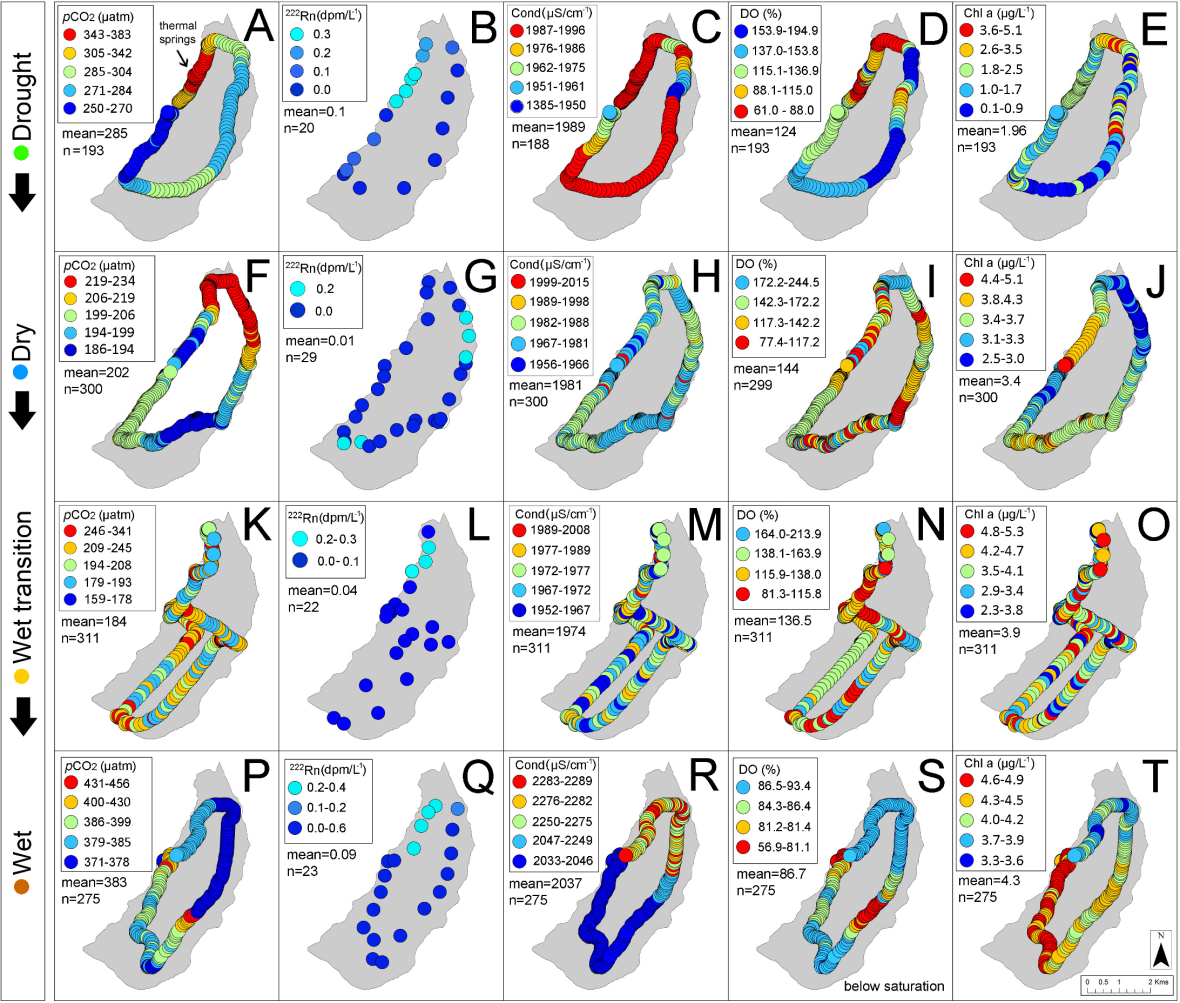
Seasonal underway measurements in Lake Batur showing the spatial distribution of *p*CO_2_ and associated parameters. ^222^Rn was measured in 1 minute intervals while *p*CO, conductivity, dissolved oxygen and chlorophyll a were measured in 10 minute intervals. Note the location of thermal springs (A) and different colour scale categories for each sampling period to highlight spatial patterns.

**Fig 5:**
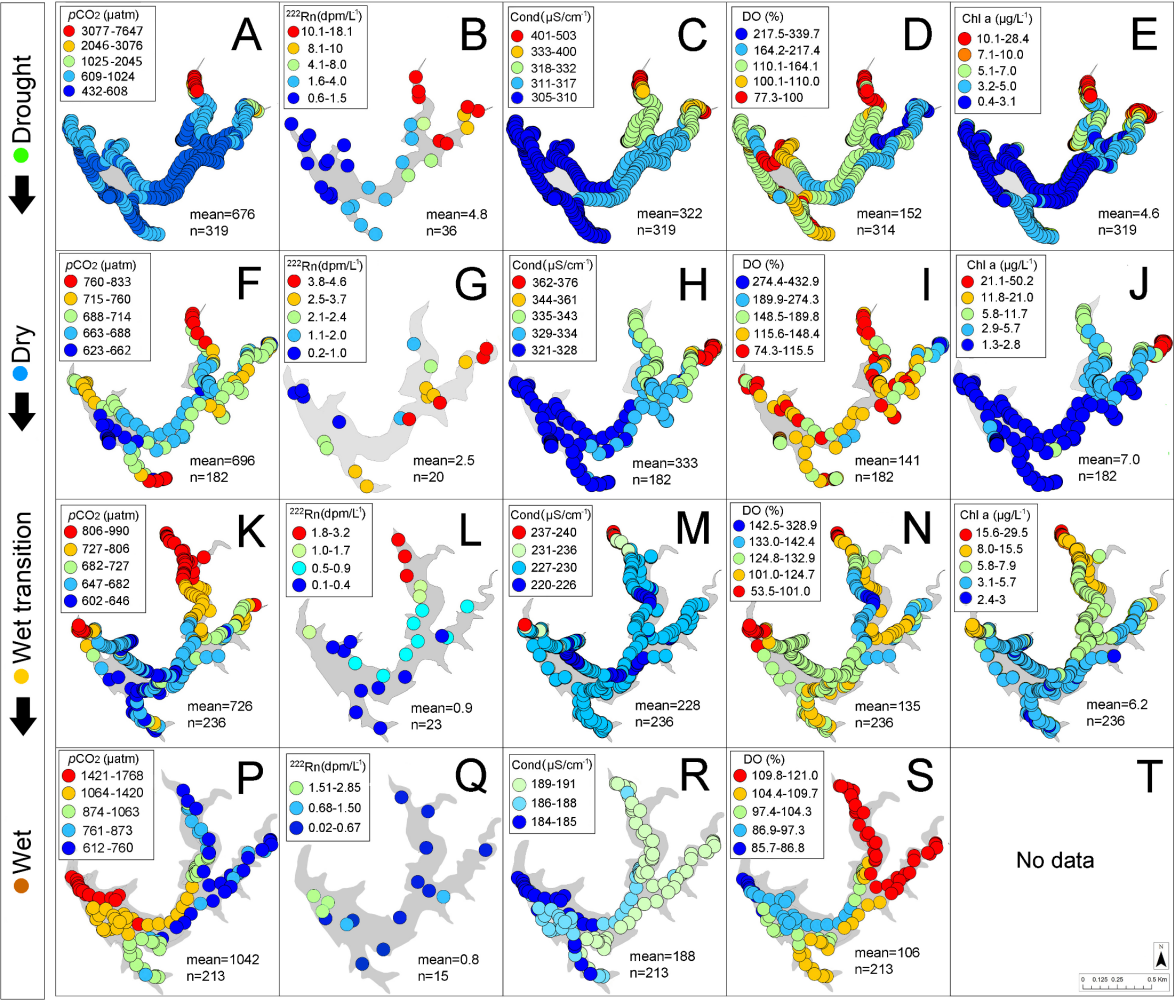
Seasonal underway measurements in Palasari Reservoir showing the spatial distribution of *p*CO_2_ and associated parameters. ^222^Rn was measured in 1 minute intervals while *p*CO, conductivity, dissolved oxygen and chlorophyll a were measured in 10 minute intervals. Note the location of a small stream (A) and different colour scale categories for each sampling period to highlight spatial patterns.

The natural lake recorded lower average temperatures (25.8°C) than the reservoir (31.9°C) (Table 3) while both ranges were similar, suggesting both have surface water temperature driven by differences in elevation (Table 1). Average annual conductivity in the natural lake was ~7-fold higher (1991.4 μS/cm) than in the reservoir (290.6 μS/cm) increasing towards dry creek bed tributaries in the reservoir only (Figs 4 and 5) while dissolved oxygen was generally supersaturated in both systems.

**Table 3:** Minimum, maximum, mean and standard deviations of underway variables (*p*CO_2_, temperature, dissolved oxygen and conductivity) over 4 seasonal sampling periods in the natural lake (Lake Batur) (left) and the reservoir (Palasari Reservoir) (right).

			Lake Batur				Palasari Reservoir				
		Drought	Dry	Wet	Wet	Average	Drought	Dry	Wet	Wet	Average
				transition					transition		
											
pCO_2_	Min	250.3	186.2	159.1	371.2	**241.7**	432.1	623.4	602.6	612.4	**567.6**
(μatm)	Max	383.3	234.3	341.4	456.1	**353.8**	7647	832.7	989.6	1768.2	**2809.4**
	**Mean**	**285.5**	**202.5**	**184.3**	**382.6**	**263.7**	**676.3**	**696.2**	**725.8**	**1041.7**	**785.0**
	St. dev ±	18.7	10.8	11.5	7.6	**12.2**	659.3	42.7	92.9	339.5	**283.6**
^222^Rn	Min	0	0	0	0	**0.0**	0.6	0.2	0.1	0	**0.2**
(dpm/L^-1^)	Max	0.3	0.2	0.3	0.4	**0.3**	18.1	4.6	3.2	2.8	**7.2**
	**Mean**	**0.1**	**0.01**	**0.04**	**0.09**	**0.1**	**4.8**	**2.5**	**0.9**	**0.8**	**2.3**
	St. Dev ±	0.1	0.04	0.07	0.14	**0.1**	5.3	1.4	0.9	0.9	**2.1**
Temp	Min	20.4	26.6	25.1	22.1	**23.6**	29.8	32.6	30.2	29.2	**30.5**
(°C)	Max	32	28.1	26.7	25.2	**28.0**	34.9	33.9	36.9	30.1	**34.0**
	**Mean**	**25.7**	**27.1**	**26**	**24.5**	**25.8**	**31.8**	**33.2**	**32.8**	**29.7**	**31.9**
	St. Dev ±	2.1	0.3	0.4	0.8	**0.9**	1.1	0.4	1.2	0.3	**0.8**
DO	Min	61	77.4	81.3	56.9	**69.2**	77.3	74.3	53.5	85.7	**72.7**
(%)	Max	194.9	244.5	213.9	93.4	**186.7**	339.7	432.9	328.9	121	**305.6**
** **	**Mean**	**124.1**	**144**	**136.5**	**86.7**	**122.8**	**151.9**	**141.4**	**135**	**105.8**	**133.5**
	St. Dev ±	32.6	31.9	30.1	4.8	**24.9**	54.7	50.7	59.7	11.1	**44.1**
Cond.	Min	1385	1979	1952.3	2033	**1837.3**	305	321	220.0	184	**257.5**
(μS/cm^-1^)	Max	1996	1984	2008.8	2289	**2069.5**	503	376	240.0	191	**327.5**
	**Mean**	**1989.5**	**1981.4**	**1973.9**	**2037**	**1995.5**	**321.9**	**333**	228.3	**187.8**	**267.8**
	St. Dev ±	104.6	6.3	7.4	1.6	**30.0**	34.6	10.4	3.3	2.4	**12.7**
Chl a	Min	0.1	2.48	2.3	3.3	**2.0**	0.4	1.3	2.4	No data	**1.4**
(μg/L^-1^)	Max	5.1	5.12	5.3	4.9	**5.1**	28.4	50.2	29.5	No data	**36.0**
	**Mean**	**1.96**	**3.43**	**3.9**	**4.3**	**3.4**	**4.61**	**6.98**	**6.2**	**No data**	**5.9**
	St. Dev ±	0.94	0.42	0.88	0.86	**0.8**	4.08	12.5	3.6	No data	**6.7**
											

**Fig 6:**
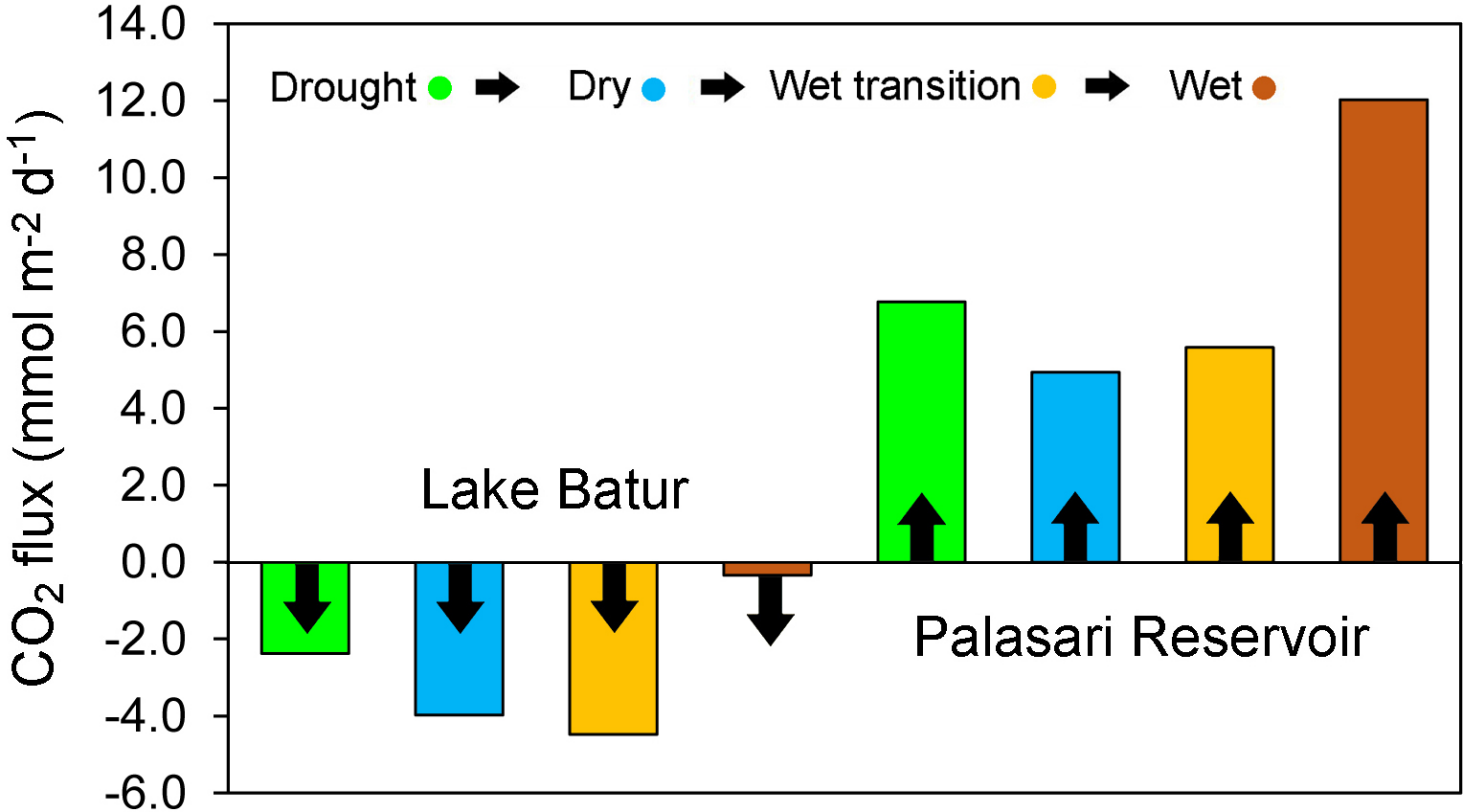
Average seasonal CO_2_ fluxes (mmol m^-2^ d^-1^) for Lake Batur (left) and Palasari Reservoir (right) with each survey period colour coded as drought (green), dry (blue), wet transition (orange) and wet (brown).

^222^Rn was ≤0.4 dpm/L^-1^ in the natural lake (Fig 4) and significantly higher in the reservoir during the drought period when it ranged from 0.6 dpm/L ^-1^ to 18.1 dpm/L ^-1^ (mean = 4.8 dpm/L^-1^). ^222^Rn decreased seasonally with increasing rainfall in the dry, wet transition and wet periods (Figs 5 and 6; Table 3).

CO_2_ was undersaturated in the natural lake with the exception of a wet period (June, 2016) where ~20% of locations were supersaturated, reaching 451 μatm (Fig 4). The reservoir remained supersaturated in CO_2_ throughout the year (Fig 5). The highest reservoir CO_2_ ranges of 432 to 7647 μatm occurred in the drought period and were ~24-fold higher than that of the natural lake range (159–456 μatm) (Table 3). CO_2_ followed the same spatial trend as ^222^Rn concentrations increasing towards the reservoir dry creek bed tributaries although no flowing streams were visible (Fig 5A).

**Fig 7:**
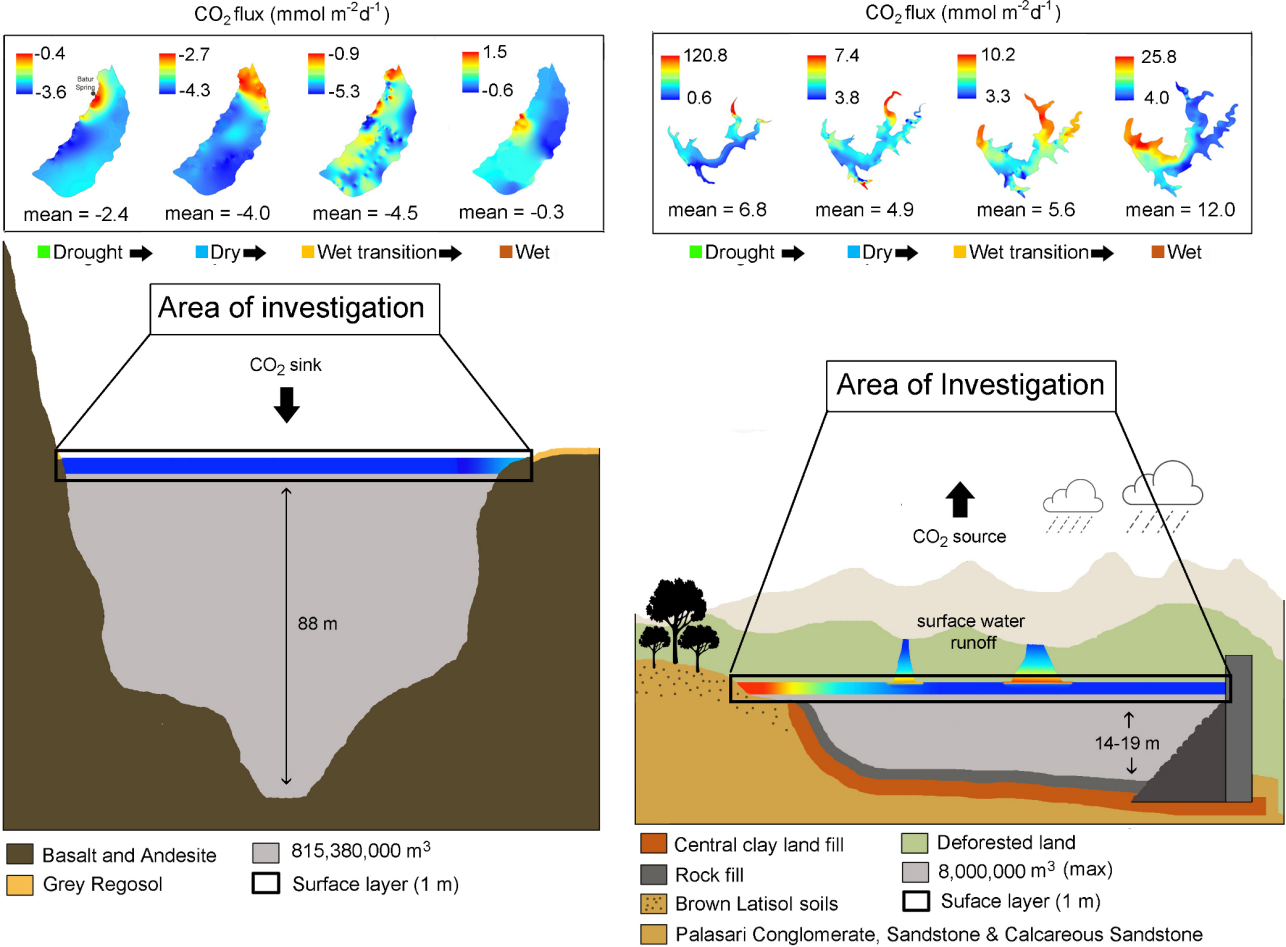
Area weighted seasonal CO_2_ fluxes (mmol m^-2^ d^-1^) averaged from the equations of six authors (Wanninkhof, 1992; MacIntyre et al., 1995; Cole & Carico, 1998; McGillis et al.,2001; Crusius and Wanninkhof, 2003; Cole et al.,2010) and interpolated using the ArcMap GIS spline with barriers method, through the drought, dry, wet transition and wet periods in Lake Batur (top) and Palasari Reservoir (bottom).

Overall the lake was a sink of atmospheric CO_2_ (average *p*CO_2_ = 263.7±12.2 μatm) with average area weighted fluxes of -2.8±0.3 mmol m^-2^ d^-1^ tending towards atmospheric equilibrium in the wet period (average = 382.6±7.6 μatm) while the reservoir was a source with an average *p*CO_2_ of 785.0±284 μatm and CO_2_ evasion of 7.3±6.7 mmol m^-2^ d^-1^ (Figs 6 and 7; Table 4). CO_2_ uptake increased in the natural lake from -2.4±0.4 mmol m^-2^ d^-1^ during the drought period to -4.5±0.3 mmol m^-2^ d^-1^ during the wet transition period, with a tendency towards atmospheric equilibrium in the wet period when the area-weighted flux was -0.3 mmol m^-2^ d^-1^.

**Table 4:** Instantaneous windspeed and CO_2_ fluxes of the 4 different transfer velocity parameterizations (see Table 2) for the natural lake (Lake Batur) and the reservoir (Palasari Reservoir) throughout the sampling period. Area weighted CO_2_ fluxes for Lake Batur (left) and Palasari Reservoir (right) and interpolated GPS point data using the Arcmap GIS spline with barriers interpolation method.

		Lake Batur						Palasarai Reservoir				
Code		Drought	Dry	Wet	Wet	Average		Drought	Dry	Wet	Wet	Average
				transition						transition		
		*CO*_*2*_ *fluxes (mmol m*^*-2*^ *d*^*-1*^*)*				* *		*CO*_*2*_ *fluxes (mmol m*^*-2*^ *d*^*-1*^*)*				
W92	Min	-4.1	-5.7	-7	-0.8	**-4.4**		0.9	6.3	5.6	5.8	**4.7**
	Max	-0.5	-3.7	-1.2	1.8	**-0.9**		212.6	12.3	16.6	37.1	**69.7**
	Mean	-3.1	-5.3	-5.8	-0.4	**-3.7**		11.4	8.2	9.3	17.4	**11.6**
	St Dev ±	0.5	0.3	0.3	0.2	**0.3**		32.2	1	2.7	9.2	**11.3**
M95	Min	-3.6	-5.1	-6.4	-0.7	**-4.0**		0.8	5.6	5	5.1	**4.1**
	Max	-0.4	-3.3	-0.9	1.7	**-0.7**		189.5	11	14.8	33.1	**62.1**
	Mean	-2.7	-4.7	-5.1	-0.4	**-3.2**		10.1	7.3	8.2	15.5	**10.3**
	St Dev ±	0.5	0.3	0.3	0.2	**0.3**		28.7	0.9	1.9	8.2	**9.9**
C&C98	Min	-4.5	-4.8	-6	-0.7	**-4.0**		0.6	3.5	2.9	4.1	**2.8**
	Max	-0.4	-3.1	-1	1.7	**-0.7**		103.9	6.7	9.6	27.3	**36.9**
	Mean	-2.7	-4.4	-5.1	-0.3	**-3.1**		6.2	4.6	5.2	12.6	**7.2**
	St Dev ±	0.4	0.3	0.3	0.2	**0.3**		15.6	0.4	1.4	6.8	**6.1**
M01	Min	-5.2	-5.5	-6.9	-0.9	**-4.6**		0.7	4	3.4	4.8	**3.2**
	Max	-0.5	-3.6	-1.2	2.1	**-0.8**		120.1	7.8	11	31.6	**42.6**
	Mean	-3.1	-5.1	-5.9	-0.5	**-3.7**		7.1	5.3	5.9	14.6	**8.2**
	St Dev ±	0.5	0.3	0.4	0.3	**0.4**		18	0.5	1.6	7.9	**7.0**
C&W03	Min	-3.5	-3.8	-4.8	-0.6	**-3.2**		0.5	2.8	2.3	3.3	**2.2**
	Max	-0.3	-2.4	-0.8	1.4	**-0.5**		82.2	5.3	7.6	21.6	**29.2**
	Mean	-2.3	-3.5	-4.1	-0.3	**-2.6**		4.9	3.6	4.1	10	**5.7**
	St Dev ±	0.4	0.2	0.2	0.2	**0.3**		12.3	0.4	1.1	5.4	**4.8**
C10	Min	-0.7	-0.8	-1	-0.1	**-0.7**		0.1	0.5	0.5	0.6	**0.4**
	Max	-0.1	-0.5	-0.2	0.3	**-0.1**		16.3	1.1	1.5	4.3	**5.8**
	Mean	-0.4	-0.7	-0.8	-0.1	**-0.5**		1	0.7	0.8	2	**1.1**
	St Dev ±	0.1	0.04	0.1	0.04	**0.1**		2.4	0.1	0.2	1.1	**1.0**
**Average**	Min	-3.6	-4.3	-5.3	-0.6	**-3.5**		0.6	3.8	3.3	4	**2.9**
**Combined**	Max	-0.4	-2.7	-0.9	1.5	**-0.6**		120.8	7.4	10.2	25.8	**41.1**
**Flux**	**Mean**	**-2.4**	**-4**	**-4.5**	**-0.3**	**-2.8**	** **	**6.8**	**4.9**	**5.6**	**12**	**7.3**
	St Dev ±	0.4	0.2	0.3	0.2	**0.3**		18.2	0.5	1.5	6.4	**6.7**
						** **						** **

## 4. Discussion

### 4.1 Contrasting CO_2_ dynamics in the natural lake and reservoir

**Fig 8:**
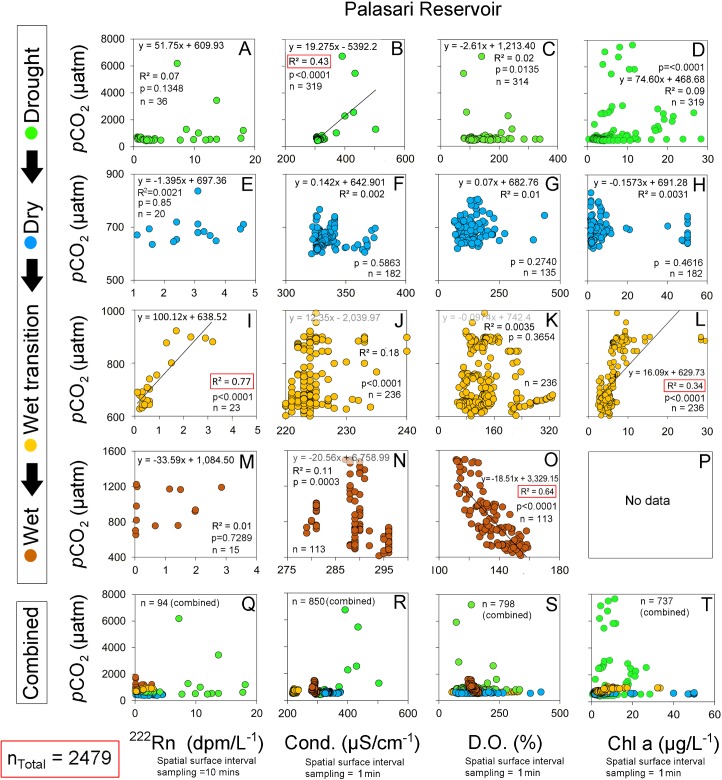
Relationships between *p*CO_2_ and ^222^Rn (groundwater tracer), conductivity, dissolved oxygen and chlorophyll a (from left to right) in reservoir (Palasari Reservoir).

We have assessed seasonal CO_2_ dynamics on a natural and artificial lake in a tropical volcanic island, building on earlier work that focused mostly on boreal and temperate regions [37]. Volcanic caldera lakes such as Lake Batur typically have high groundwater recharge rates due to fracture-induced permeability [38], and small overall surface-groundwater interactions [39]. In contrast, artificial shallow lakes such as Palasari Reservoir typically have more pronounced terrestrial sources that may stimulate productivity [40]. This is highlighted in the reservoir’s elevated ranges of surface water *p*CO_2_ with spatially variable measurements of surface water conductivity, dissolved oxygen and chlorophyll a (Figs 5 and 8; Table 3). This reflects seasonal rainfall influences with larger catchment sizes and watershed-to-reservoir ratios, when compared to the smaller catchments found in caldera lakes [41]. This is strongly shown in the differences in CO_2_ concentrations within the natural lake and reservoir during the sampling period (Figs 4 and 5; Table 3). Although higher *p*CO_2_ values were measured in the shallow (< 2m) near-shore zones in the reservoir, this did not result in large emissions when taking into account area weighted *p*CO_2_ measurements (Fig 7).

For large and small systems such as Lake Batur and Palasari Reservoir, respectively, differences in CO_2_ may be influenced by depth (due to the volume-to-sediment surface ratios), catchment area and lake area ratios (2:1 and 420:1 respectively; Table 1). These ratios exert strong influences over delivery of terrestrial organic matter and water chemistry [42]. For example, a study of 82 boreal lakes (areas = 0.04–1540 km2; max depth = 1–93 m) in Finland found that lake area and depth are important predictors of CO_2_ evasion with higher emissions found in small, shallow lakes [43] In addition, nutrient delivery results from variations of surface water inflow dependent on regional rainfall regimes and geographic location. These traits are reflected in the lower average *p*CO_2_ concentrations in the large, deep natural lake when compared to the small, shallow reservoir.

**Fig 9:**
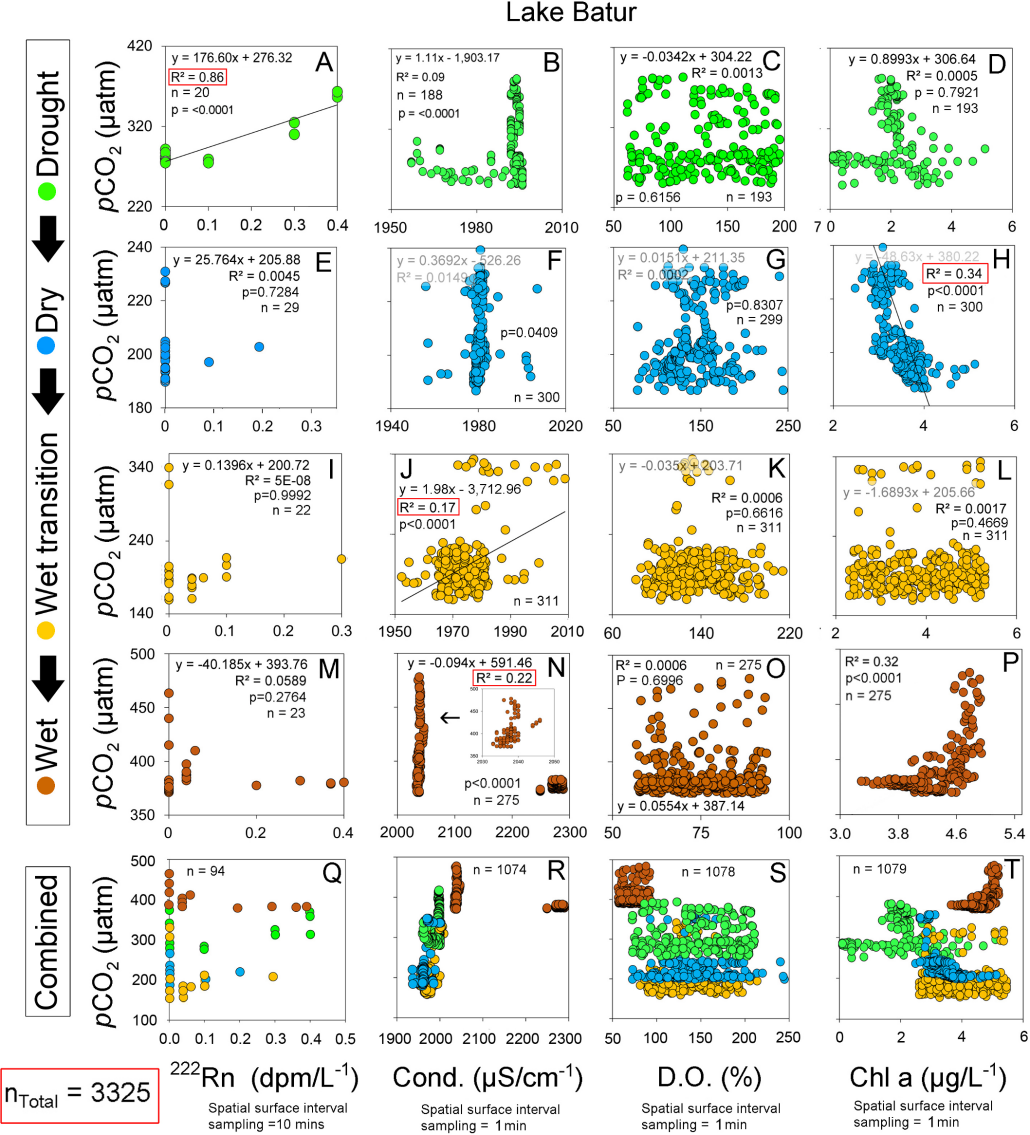
Relationships between *p*CO_2_ and ^222^Rn (groundwater tracer), conductivity, dissolved oxygen and chlorophyll a (from left to right) in the natural lake (Lake Batur).

Correlations between CO_2_ and conductivity, dissolved oxygen and chlorophyll a were similar in the deeper reservoir and in the natural lake (Figs 8 and 9). The strongest reservoir *p*CO_2_ correlations with conductivity, DO and chlorophyll a were in the drought period (r^2^ = 0.77; p<0.0001), wet period (r^2^ = 0.64; p<0.0001) and wet transition period (r^2^ = 0.34; p<0.001) respectively. However, the reservoir near-shore zone had more linear correlations typical of other lotic systems [44] but represented a minimal spatial area (see Fig 5A and Fig 8). In the natural lake, chlorophyll a accounted for 34% and 32% of the CO_2_ variability in the dry and wet period, respectively (Fig 9H and 9P). However, this relationship reversed from the drought to wet period. This reflected similarities with the reservoir which also showed a positive correlation between CO_2_ and chlorophyll a (r^2^ = 0.34) in the wet transition period (Fig 8L).

Several previous studies have used ^222^Rn as a tracer in inland waters [28, 44, 45]. By simultaneously measuring the groundwater tracer ^222^Rn and *p*CO_2_, we found similarities to previous estuarine studies where radon followed CO_2_ distributions [46, 47] (Figs 5 and 8). Groundwater inputs were apparently negligible in the natural lake (average ^222^Rn 0.06 dpm/L^-1^; Table 3) and was not considered as a significant driver of CO_2_ dynamics. In the reservoir, ^222^Rn decreased from the drought to wet periods. (Figs 5 and 8). Decreased wet period groundwater flow may be due to high surfacewater inputs with *p*CO_2_ dynamics linked to this surfacewater loading [48]. Overall, our observations imply a stronger groundwater influence in the reservoir than in the natural lake.

### 4.2 Rainfall as a driver of CO_2_ in tropical and temperate systems

Short, intense rainfall events, which are common in Bali’s wet season, have recently been acknowledged as important pathways of terrestrial carbon loading to in inland waters [44, 48]. Artificial reservoirs and natural lakes receive CO_2_ produced and derived from their catchment areas as a result of rainfall events when large amounts of carbon are rapidly transported to these waterbodies [49]. Many studies have reported correlations between atmospheric CO_2_ fluxes and rainfall events [29, 50] with significant amounts of terrestrial CO_2_ delivery to lake waters during these events [1].

**Fig 10:**
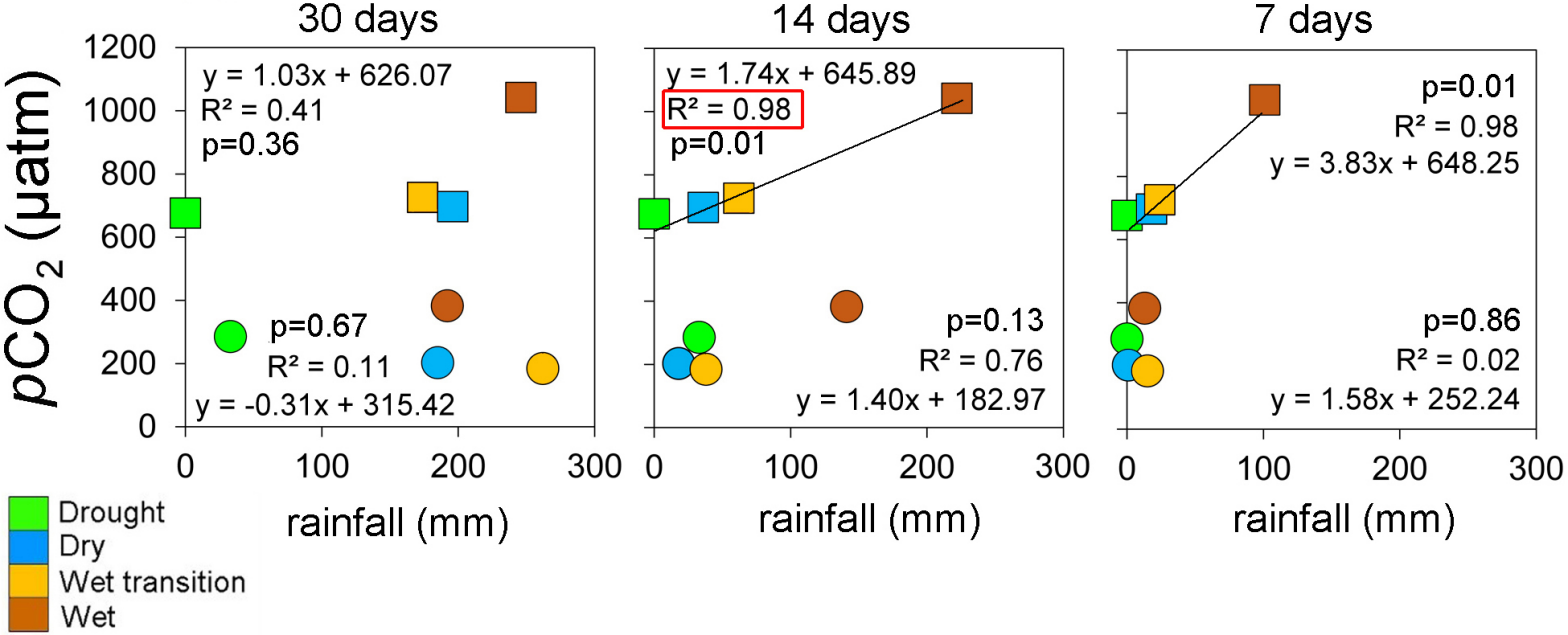
Rainfall (30, 14 and 7 days prior) and *p*CO_2_ dynamics in Lake Batur (circles) and Palasari Reservoir (squares) indicating differences in average *p*CO_2_ in the confined and open lake in drier periods and elevated average *p*CO_2_ in both systems after heavy rainfall.

In spite of the small sample size, we found a correlation between CO_2_ and antecedent rainfall in the reservoir and lake (Fig 10). Rainfall events have previously been reported to deliver large amounts of particulate and dissolved organic carbon into aquatic systems [51, 52]. Higher seepage of CO_2_ enriched groundwaters in the wet transition and wet sampling periods implies that wetter conditions lead to higher groundwater input due to a larger hydraulic head. In small lakes in northern Europe, CO_2_ increased in the soil and lake following a significant rainfall event (61 mm). Terrestrial flushing was reflected in the high surface water CO_2_ concentrations, with *p*CO_2_ increasing from 1800 to 4370 μatm soon after the rain event [27].

In the reservoir, the effects of rainfall are emphasised by the decoupling of *p*CO_2_ and ^222^Rn which was elevated near a stream in the northern area (Fig 5). Tropical reservoir studies have reported rainfall events which load high amounts of terrestrial CO_2_ into receiving waters [27, 29, 49] by increased river discharge [53] and carbon rich terrestrial inputs as a result of soil erosion [51]. Tropical regions in particular are prone to high CO_2_ terrestrial loading as a result of episodic heavy rainfall events, pronounced wet seasons and high surface water temperatures. The low lying topography and plantations in the northern area of the reservoir may support more groundwater interactions (Fig 5A and Table 1). This is reflected in Fig 5 and supported by Fig 8, with outliers representing relatively small areas but reflecting the groundwater (^222^Rn) dominated characteristics of the reservoir near-shore zone.

**Fig 11:**
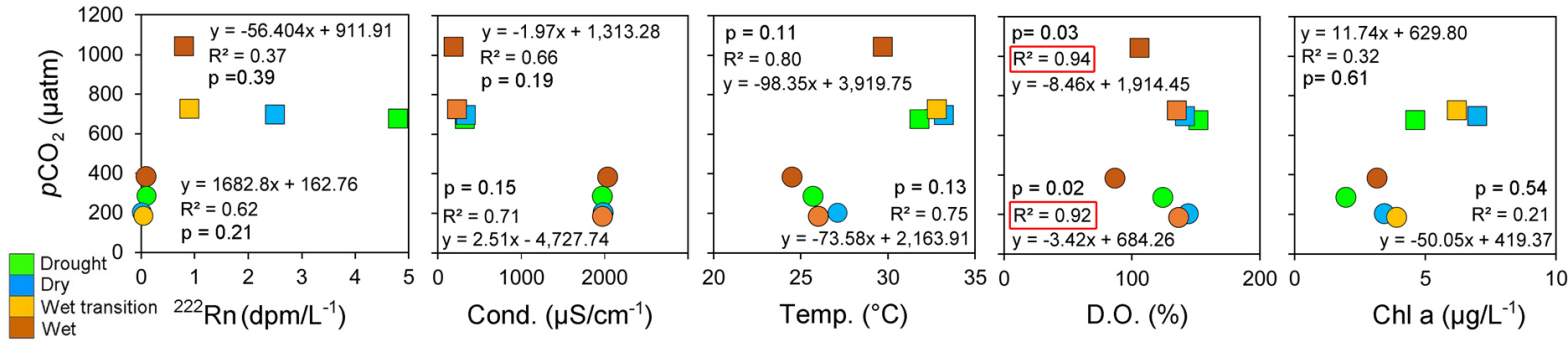
Seasonal averages for the drought, wet, wet transition and wet periods for Lake Batur (circle) and Palasari Reservoir (square), indicating relationships between CO_2_ and ancillary variables.

A recent study showed that water-to-air CO_2_ fluxes in Brazilian lakes were significantly enhanced in heavy rainfall events, recording 28.5 ± 6.0 mmol CO_2_ m^−2^ d ^−1^ in dry periods and 245.3.1 ± 51.5 mmol CO_2_ m^−2^ d ^−1^ shortly after the heavy rainfall event. The increased inputs of CO_2_ following periods of high rainfall were believed to be derived from increased inputs of CO_2_ from groundwater to the lakes, resulting in an ~10-fold increase in lake *p*CO_2_ [29]. Similarly, the natural lake from Bali sequestered atmospheric CO_2_ throughout each sampling campaign but *p*CO_2_ increased and approached atmospheric equilibrium in the wet period as a result of heavy rainfall events (Figs 10 and 11). The depth of the natural lake (mean = 50.8; max = 88 m; Table 1) may dilute elevations in *p*CO_2_ as a result of terrestrial carbon inputs or groundwater inputs. Most previous lake investigations are from shallower systems [4, 37]. There is a paucity of data on CO_2_ dynamics from such water bodies as the dominant caldera lakes in tropical volcanic regions such as Indonesia. Therefore, our observations may help to fill a gap in global CO_2_ observations in lakes.

### 4.3 Implications

Due to uneven spatial and temporal distribution of rainfall and river flow, reservoir construction is becoming increasingly important in regions of fast population growth such as Southeast Asia. We speculate that the accelerated construction of reservoirs [54, 55] and population growth [55] will increase the contribution of Southeast Asia inland waters to the global carbon budget. In Bali, the current reservoir capacity has a ratio of storage per capita of only 63.5 m^3^ which is inadequate to cope with the increasing water demand (Direktorat Jenderal Sumber Daya Air: http://sda.pu.go.id/). Bali’s local population of 4,200,000 in 2012 had a water demand of 229,950,000 m^3^ yr^-1.^ This does not take into account irrigation water for rice, agriculture, industry and tourism growing 20% between 2015 and 2016. Assuming future reservoir construction will supply water demand, reservoirs may become a more significant regional CO_2_ source that will need to be managed effectively. In 2015, Indonesia had 6 reservoirs under construction, 6 in negotiation stages and 7 in design stages, not taking into account current small reservoir construction (www.narbo.jp/).

Lakes and reservoirs in tropical regions, as a result of stable temperature and light, have been recently reported to have lower seasonal variations of biological activity when compared to boreal and temperate counterparts [56]. For example low variations of seasonal CO_2_ concentrations in a tropical lake (Lake Kivu, East Africa) were linked to tropical climate and partly associated with minimal water temperature variations [56]. Predicted increases in both tropical monsoonal temperatures and reservoir construction may increase the inland water contribution to the global carbon budget.

Tropical lakes and reservoirs are under-represented and comprise only 1.5% of the global dataset (n = 7939) of CO_2_ emissions (Raymond et al., 2013). While tropical lakes may be responsible for 34% of the global atmospheric CO_2_ fluxes from inland waters, they cover only 2.4% of the global lake area [1]. Lake ranges and averages of *p*CO_2_ were found to be amongst the highest in tropical regions. Rainfall and temperature appeared to be a strong controls over *p*CO_2_ in our study. The predicted temperature increase would increase bacterial metabolism resulting in more organic carbon respiration. Due to poor representation in global datasets, constraining CO_2_ fluxes in tropical lakes and reservoirs is particularly important [4].

## 5. Conclusions

Our observations in Bali revealed that antecedent rainfall seems to be a major control on seasonal CO_2_ distributions in both the lake and reservoir. The spatial distribution of *p*CO_2_ was driven primarily by autochthonous processes (water column metabolism) in the deep lake, and allochthonous processes (groundwater seepage) in the shallow reservoir. Overall, the natural lake was an atmospheric CO_2_ sink, while the reservoir was releasing CO_2_ to the atmosphere. We speculate that the predicted increase in reservoir area in tropical regions may increase CO_2_ fluxes to the atmosphere and partially offset the sink provided by deep, volcanic, natural lakes. Site specific carbon investigations are needed to monitor inland waters on a regional scale. Due to the rapid expansion of reservoir construction, particularly in tropical regions, it may be necessary to develop long term monitoring programs that capture reservoir evolution and infilling process as well as large scale comparative studies already available for better studied northern hemisphere lakes.
