# Correction: Carbon dioxide dynamics in a lake and a reservoir on a tropical island (Bali, Indonesia)

**DOI:** 10.1371/journal.pone.0200948

**Published:** 2018-07-12

**Authors:** The PLOS ONE Staff

## Notice of republication

This article was republished on June 26, 2018, to correct an error in the ORCID iD for the corresponding author. The publisher apologizes for the error. Please download this article again to view the correct version. The originally published, uncorrected article and the republished, corrected articles are provided here for reference.

## Supporting information

S1 File Originally published, uncorrected article. (PDF)

S2 File Republished, corrected article. (PDF)
