# Supplementary material for: Correction: Carbon dioxide dynamics in a lake and a reservoir on a tropical island (Bali, Indonesia)
Source: PLoS One. 2018 Jul 12;13(7):e0200948. doi: 10.1371/journal.pone.0200948 (PMC6042792; doi:10.1371/journal.pone.0200948)
Supplement: S1 File — (PDF) [file pone.0200948.s001.pdf]

PLOS ONE

RESEARCH ARTICLE

Carbon dioxide dynamics in a lake and a reservoir on a tropical island (Bali, Indonesia)

Paul A. Macklin[1,2]*, **I. Gusti Ngurah Agung Suryaputra**[3], **Damien T. Maher**[1,2], **Isaac R. Santos**[1,2]

1 National Marine Science Centre, Southern Cross University, Coffs Harbour, New South Wales, Australia, **2** School of Environment, Science and Engineering, Southern Cross University, Lismore, New South Wales, Australia, **3** Department of Analytical Chemistry, Universitas Pendidikan Ganesha, Singaraja, Bali, Indonesia

* paul.macklin@gmail.com

Abstract

Water-to-air carbon dioxide fluxes from tropical lakes and reservoirs (artificial lakes) may be an important but understudied component of global carbon fluxes. Here, we investigate the seasonal dissolved carbon dioxide (CO_2) dynamics in a lake and a reservoir on a tropical volcanic island (Bali, Indonesia). Observations were performed over four seasonal surveys in Bali's largest natural lake (Lake Batur) and largest reservoir (Palasari Reservoir). Average CO_2 partial pressures in the natural lake and reservoir were 263.7±12.2 μatm and 785.0 ±283.6 μatm respectively, with the highest area-weighted partial pressures in the wet season for both systems. The strong correlations between seasonal mean values of dissolved oxygen (DO) and pCO_2 in the natural lake ($r^2 = 0.92$) suggest that surface water metabolism was an important driver of CO_2 dynamics in this deep system. Radon (^{222}Rn, a natural groundwater discharge tracer) explained up to 77% of the variability in pCO_2 in the shallow reservoir, suggesting that groundwater seepage was the major CO_2 driver in the reservoir. Overall, the natural lake was a sink of atmospheric CO_2 (average fluxes of -2.8 mmol m^{-2} d^{-1}) while the reservoir was a source of CO_2 to the atmosphere (average fluxes of 7.3 mmol m^{-2} d^{-1}). Reservoirs are replacing river valleys and terrestrial ecosystems, particularly throughout developing tropical regions. While the net effect of this conversion on atmospheric CO_2 fluxes remains to be resolved, we speculate that reservoir construction will partially offset the CO_2 sink provided by deep, volcanic, natural lakes and terrestrial environments.

Introduction

Lakes and reservoirs cover 2.2% of the global surface area [1]. Although relatively small in aerial extent, lakes play a significant and increasingly important role in the global carbon cycle [2, 3]. Estimates of the global net CO_2 flux for lakes and reservoirs is ~0.3 Pg C yr^{-1} (range 0.06 to 0.84 Pg C yr^{-1}) but there are uncertainties in the number and area of small lakes [1]. With climate change, it is likely that there will be a global changes in lake abundance. It is predicted that there will be losses of natural lakes in regions where the climate is becoming drier [4] and increases in reservoir construction [5] in regions with rapidly expanding populations such as Southeast Asia [4]

Check for updates

OPEN ACCESS

Citation: Macklin PA, Suryaputra IGNA, Maher DT, Santos IR (2018) Carbon dioxide dynamics in a lake and a reservoir on a tropical island (Bali, Indonesia). PLoS ONE 13(6): e0198678. https://doi.org/10.1371/journal.pone.0198678

Editor: Xiujun Wang, Beijing Normal University, CHINA

Received: June 24, 2017

Accepted: May 23, 2018

Published: June 11, 2018

Copyright: © 2018 Macklin et al. This is an open access article distributed under the terms of the Creative Commons Attribution License, which permits unrestricted use, distribution, and reproduction in any medium, provided the original author and source are credited.

Data Availability Statement: The data underlying this study have been uploaded to figshare and are accessible using the following link: https://figshare.com/articles/S1_Supporting_Information_Water-air_CO2_flux_Dataset/5899741.

Funding: Paul Anthony Macklin was supported by the Australian Government Research Training Program Scholarship. We acknowledge funding support from the Australian Research Council (DE140101733, DE150100581, and LE120100156) that partially funded IRS and DTM salaries and the portable analytical instrumentation.

Competing interests: The authors have declared that no competing interests exist.

Lakes and reservoirs modify freshwater flows of inland waters and alter CO_2 fluxes by retaining ~50% of the global carbon transported to the oceans [4]. Estimates of global reservoir numbers have varied significantly from 25, 410 to 515,149 [6, 7], the latter study including smaller reservoirs. In spite of this variability, reservoirs are important compared to natural lakes (n = 304 million) with an estimated 277 million small lakes (0.001–0.01 km^2) [7]. Millions of smaller reservoirs (<0.5 km^2; [8]) are not accounted for in global carbon budgets, and reservoir construction is increasing [8] resulting in more terrestrial carbon entering and becoming trapped in lakes and reservoirs, and changes in landscape CO_2 emissions [9]. These shifts in CO_2 emissions are further compounded by predicted increases in extreme weather events such as flood and droughts [10] as well as watershed degradation and weathering [11, 12].

Global assessments of CO_2 fluxes from lakes and reservoirs are not evenly distributed with temperate and boreal zones such as Europe and Northern America largely over-represented [13]. Tropical systems [14] and the southern hemisphere [15] are under-represented. Tropical systems comprise ~40% of the global surface area of reservoirs. However, ~70% of CO_2 fluxes from reservoirs are thought to originate in tropical regions [6]. As a result, tropical reservoirs are recognised as disproportionally large sources of CO_2 to the atmosphere [6, 16]. Compared to temperate lakes, higher water temperatures in tropical regions result in higher organic matter decomposition rates, resulting in higher CO_2 production and emissions than their temperate counterparts [4, 6].

CO_2 dynamics in both lakes and reservoirs are often driven by a combination of internal processes such as photosynthesis and respiration, as well as allochthonous inputs [17]. Allochthonous sources of CO_2 include weathering, soil organic matter and terrestrial root respiration [18, 19] and precipitation of carbonate or silicate minerals [20]. Surface water runoff [21, 22] and groundwater discharge [19, 23–25] can directly deliver terrestrial organic matter to aquatic systems, which is subsequently stored in lake sediments, exported downstream, remineralised or released to the atmosphere [4]. Since groundwater is often highly supersaturated in CO_2 when compared to surface waters and the atmosphere [26], several recent studies identified groundwater seepage as a major conduit of CO_2 to lakes [27–29].

Here, we contribute to filling knowledge gaps of inland water CO_2 dynamics in tropical regions, where there are fewer data on CO_2 outgassing rates and factors controlling this efflux than the more comprehensively studied temperate regions. We measured pCO_2 and estimate fluxes at the water-air interface along with potential drivers in a natural lake and a reservoir in Bali, Indonesia, one of the world's fastest growing tourist economies. Although Indonesia has been identified as having a high potential for groundwater recharge [30], comprehensive studies including groundwater-derived CO_2 seepage in Indonesian lakes have not been conducted to date. We hypothesize that groundwater seepage may release CO_2 to surface waters and that CO_2 concentrations will be higher in the wet season due to a relative increase in groundwater flow. Seasonal surveys of radon (a natural groundwater discharge tracer) and CO_2 are used to test this hypothesis.

2. Material and methods

2.1 Area description

Indonesia has 521 natural lakes and over 100 reservoirs which cover ~ 21,000 km^2 [31]. Bali Province is bounded by the Java Sea, the Lombok Strait, the Indian Ocean and the Bali Strait. It has 8 groundwater basins, 1273 springs, 4 lakes, 4 reservoirs, 5 ponds, and ~162 rivers (www.blh.baliprov.go.id/). Utilisation of rivers as a water source is widely unviable as the flow is intermittent, with <11% of the rivers flowing in the dry season (IDEP, 2009). Historically, Bali experiences a dry season from May to September, a transition season in October, wet

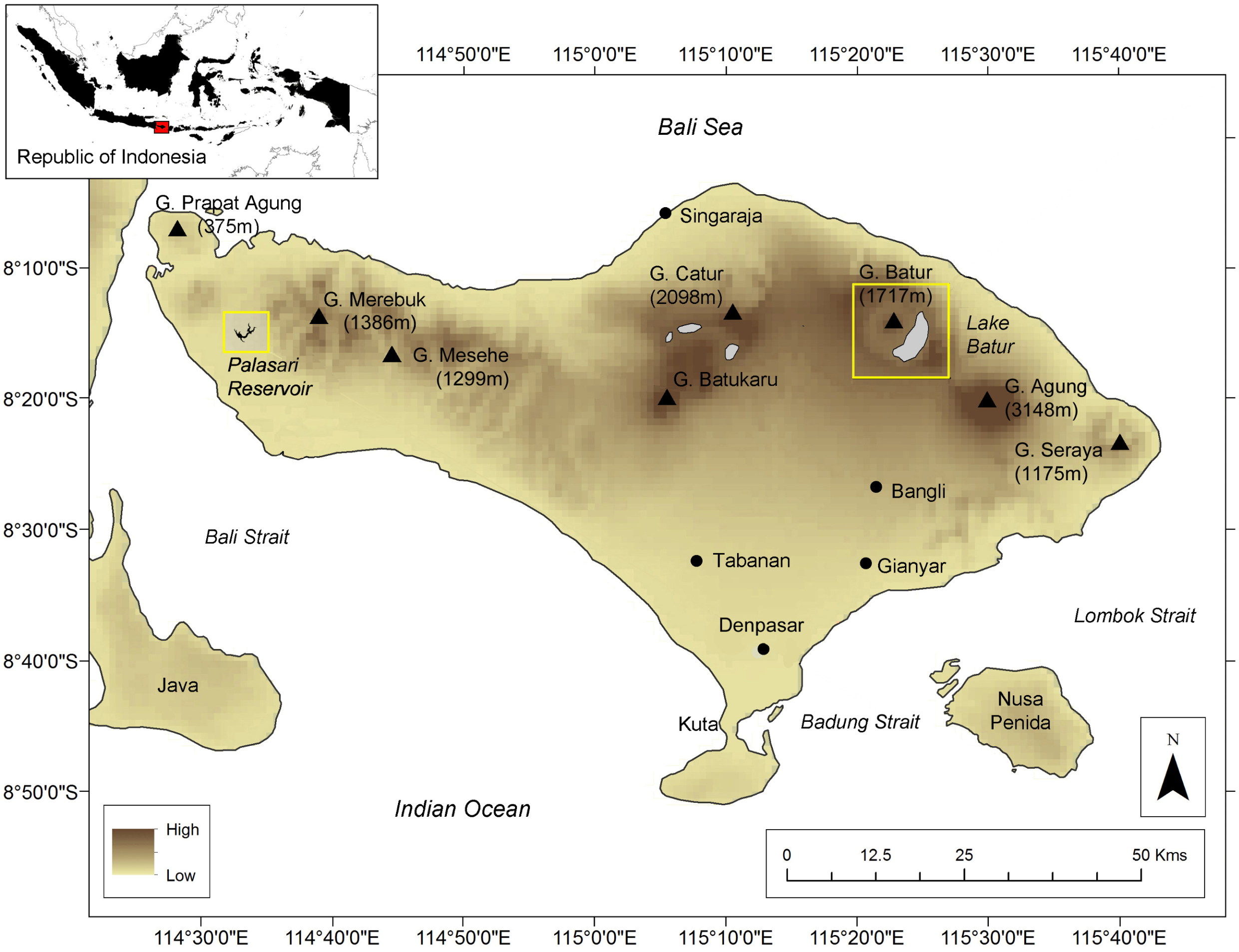

Fig 1. Palasari Reservoir (left) and Lake Batur (right) study sites (highlighted by yellow boxes) on Bali Island, Indonesia.

https://doi.org/10.1371/journal.pone.0198678.g001

season from October to April followed another transition season in March. This paper focuses on two systems on the island of Bali: Palasari Reservoir (area = 10, 056 m^2; mean depth = 16.4 m) and Lake Batur (area = 17, 180, 000 m^2; mean depth = 50.8 m) (Fig 1 and Table 1). To our knowledge, these are the first observations of CO_2 in Bali's lakes and reservoirs.

Lake Batur is Bali's largest and deepest natural lake (Fig 1). It is a confined active caldera lake formed in the depression of the collapsed volcano walls of Mt Batur with a small watershed/lake area ratio (~6:1). Topography is undulating lowlands to Mt Batur (north), and steep hills and crater walls (north, east and south). The geology is comprised of old Buyan-Bratan and Batur volcanics with basalt to basaltic andesite lavas and pyroclastic deposits underlying, and inter layered Batur Ignimbrite, (permeable when fractured with a secondary opening) and Grey Regosol soils which are vulnerable to soil erosion. The walls of the lake drop steeply to a maximum depth of 88 m, with a narrow littoral zone (Table 1) and diurnal microstratifactions and thermal and chemostratifications. Inflows to the lake includes Batur Spring, deep groundwater springs in the pyroclastic flow slope and rainfall. Small scale settlements, agriculture, aquaculture, geothermal springs and the Mt. Batur pyroclastic flow slope are found on the west side of the lake.

Palasari Reservoir is Bali's largest reservoir, located on the west coast of Bali, ~6 km downstream of Mt Sangiang (1,004 m a.s.l.). The geology of the region is dominated by the

Table 1. Morphometric and hydrological characteristics of Lake Batur and Palasari Reservoir.

Site	Units	Lake Batur	Palasari Reservoir
Latitude		8.25888˚S	8.25310˚S
Longitude		115.40825˚E	114.54920˚E
Fieldtrip dates	Drought	22/11/2015	12/10/2015
	Dry	06/01/2016	07/01/2016
	Wet transition	14/03/2016	18/03/2016
	Wet	24/06/2016	29/06/2016
Regional soils		Grey Regosol	Brown Latisol and Litosol
Lake level	m a.s.l.	1017	90
Watershed area (Wa)	m^2	105,350,000	4,230,000
Lake area (La)	m^2	17,180,000	10,056
Watershed area-lake area ratio		6.13	420.64
Max. depth	m	88	14–19 (Range in depths between surveys)
Mean depth	m	50.8	16.4
Water volume	m^3	815,380,000	8,000,000 (max)
Rainfall (sampling year)	mm/yr^{-1}	1637.0	1716.6
Rainfall (historical average)	mm	2703.6	1731.1
Lake perimeter	km	21.4	10.7
Mixing type		Monomictic	Meromictic<br>Polymictic
Location permeability	cm/sec	NA	10^{-2}–10^{-5}

https://doi.org/10.1371/journal.pone.0198678.t001

quaternary Palasari Formation which includes Palasari conglomerate, sandstone, calcareous sandstone and limestone reef. The topography is low-lying hills with regional soils dominated by Brown Latisol (http://ppsp.nawasis.info/) which are highly permeable with vegetation cover. Without vegetation cover the soil is vulnerable to erosion and rapidly becomes impermeable. The Palasari Reservoir has a large watershed area-lake area ratio (~420:1) with surrounding land use dominated by small scale agriculture. Upstream of the reservoir is protected forest with a short (<5 km) topographic transition from hilly to mountainous terrain. The reservoir is a 27 year old, rock fill type dam with a central clay land fill core of 40 m. It functions as flood control and supplies irrigation water for ~13 km^2 of rice fields downstream. Although there may be receiving inflow from the Sangiang Gode and Palareja Rivers, during the sampling period there was no notable surface water inflow into and out of the system (Table 1).

2.2 Approach and methods

We performed 4 seasonal surveys in Lake Batur and Palasari Reservoir using automated instrumentation (Fig 2). Instrumentation was installed on a small research vessel driven at 4–6 km/h to produce high spatial resolution sampling. The vessel was stopped or slowed down at sites of high interest such as areas where the landscape was modified, near stream inlets, around visible changes in nearshore vegetation, and large transitions in water depth. Location was logged continuously by a Garmin GPS72 or Maverick Pro 2.61 Android GPS.

Water column pCO_2 and ^{222}Rn concentrations were measured from a depth of ~1 meter using a portable Li-820 CO_2 detector (calibrated with 0, 400 and 10 000 ppm standards) and a radon-in-air monitor calibrated prior to deployment by the manufacturer (RAD7, Durridge; Fig 2). The detectors were connected with a closed-air-loop and an equilibrator spray chamber [26, 32] with the air stream dried with a desiccant column of Drierite. Water was continually pumped from a submersible bilge pump at about 3 L/min^{-1} into a shower head gas

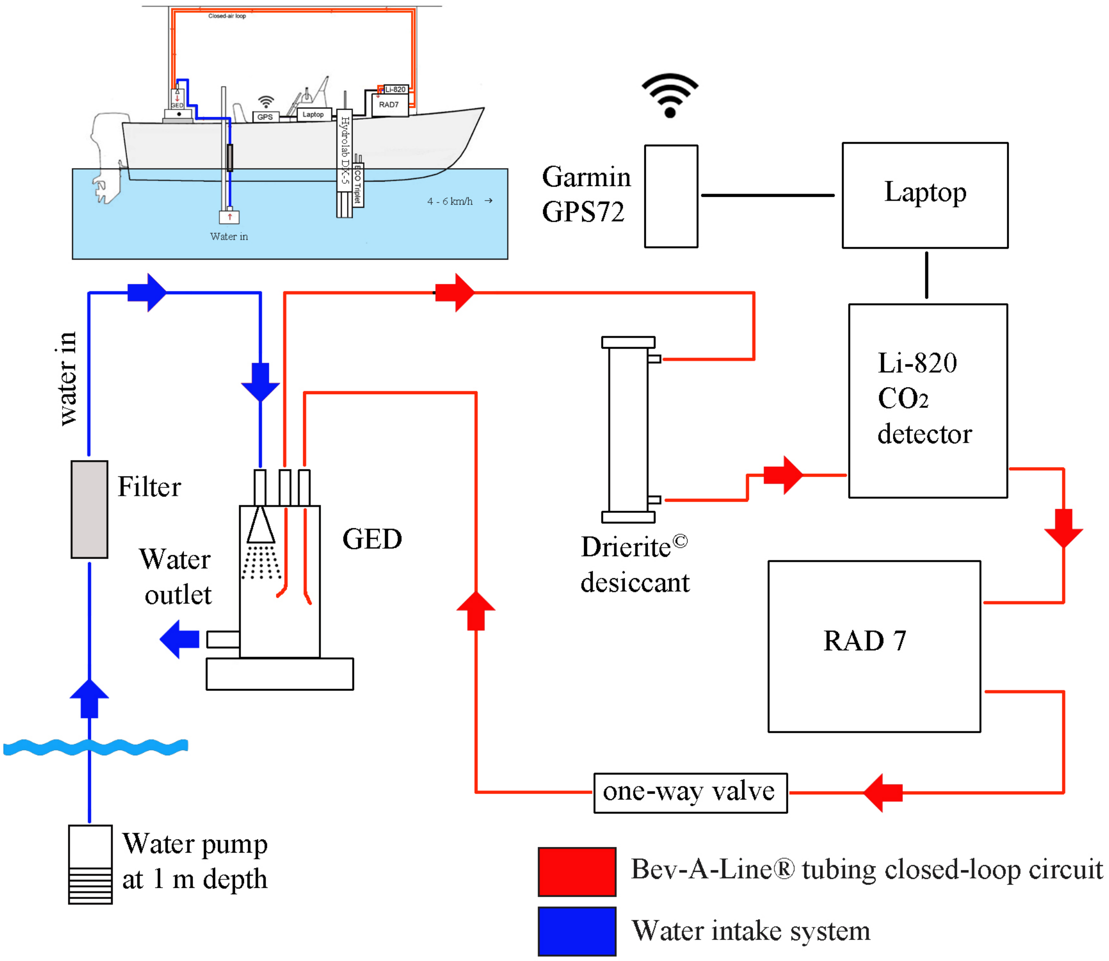

Fig 2. Coupled radon and carbon dioxide system schematic layout [26].

https://doi.org/10.1371/journal.pone.0198678.g002

equilibration device (GED). A closed-air-loop was created between the GED and gas detectors which measure the gas concentrations in the air stream. Air was pumped into the RAD7 radon detector at 1 L min^{-1} by the RAD7 internal pump. The dissolved gas concentrations were determined by the gas solubility and temperature [33, 34].

Measurements of temperature, conductivity and dissolved oxygen were undertaken using a Hydrolab DS-5 multiparameter water quality sonde, calibrated prior to each deployment at 1 min intervals to measure pH (± 0.02 units), salinity (± 0.02 ppt), dissolved oxygen (± 0.2 mg L-1), and water temperature (± 0.10˚C). pH was calibrated with 4, 7 and 10 buffer solutions (NBS scale) while conductivity was calibrated with deionised water and a 1413 μS cm^{-1} standard. Chlorophyll a was measured at 1 minute intervals with a WETlabs Eco triplet fluorometer equipped with a copper brush wiper to prevent biofouling of sensors and calibrated by the manufacturer using quinine dehydrate. Meteorogical data was sourced from Denpasar Ngurah Rai Weather Station 972300 (S08.749; E115.167).

The CO_2 flux across the water–air interface was calculated according to Wanninkhof (1992) [35]:

$$F_{CO2} = k^{*}K_{H}^{*}\Delta pCO_{2}, \quad (1)$$

Table 2. Six wind-speed based parameterization formulas with respective authors, where k is the transfer velocity (cm h^{-1}), u is the wind speed (ms^{-1}) at a height of 10 m and Sc is the Schmidt number of CO_2 at in situ temperature and salinity.

Authors		Formula	Code	Ecosystem
Wanninkhof (1992) [38]	(1)	$k = 0.31u_{10}^{2}$ $(Sc/660)^{-0.5}$	W92	Lake
MacIntyre et al. (1995) [57]	(2)	$k = 0.45u_{10}^{\ 1.6}$ $(Sc/600)^{-0.5}$	M95	Lake
Cole & Carico (1998) [58]	(3)	$k = 2.07 + 0.215u_{10}^{\ 1.7}$ $(Sc/600)^{-0.5}$	C&C98	Lake
McGillis et al. (2001) [59]	(4)	$k = 3.3 + 0.026u_{10}^{\ 3}$ $(Sc/600)^{-0.5}$	M01	Lake
Crusius and Wanninkhof (2003) [60]	(5)	$k = 0.168 + 0.228u_{10}^{2.2}$ $(Sc/600)^{-0.5}$	C&W03	Lake
Cole et al. (2010) [61]	(6)	$k = 0.497 + 0.0064u_{10}^{1.8}$ $(Sc/600)^{-0.5}$	C10	Lake

https://doi.org/10.1371/journal.pone.0198678.t002

where k is the CO_2 gas transfer velocity, K_H is the solubility of CO_2 [33] and ΔpCO_2 is the difference between the partial pressure of pCO_2 in water and air. To calculate k, we used the average of six parameterizations to provide a reasonable range in evasion rate estimates (Table 2). Positive values represent a water-to-air CO_2 flux and negative values represent an air-to-water flux. Water-to-air CO_2 fluxes were calculated by using five minute sampling times for pCO_2 and average annual windspeeds to reduce wind bias for the natural lake and reservoir, respectively. Integrated aereal CO_2 fluxes were calculated using the Spline-with-Barriers method [36] to prevent bias related to different research vessel speeds and time spent stationary.

Permits and permissions for Palasari Reservoir and Lake Batur were provided by the Indonesian Foreign Research Permit Secretariat, Ministry of Research, Technology and Higher Education of the Republic of Indonesia (RISTEKDIKTI), the Directorate General of Water Resources, the Indonesian Ministry of Public Works (DGWRD) and the Governor of Bali, I Made Mangku Pastika. Field studies did not involve endangered or protected species.

3. Results

Both study sites experienced an extended drought period during the 2015 dry season (Fig 3) with no rainfall recorded 3 months prior to initial sampling in November 2015. In contrast the 2016 dry season (May-September) recorded significantly more rainfall than historical averages. This created a sampling period with initial dry conditions transitioning to wetter conditions in both systems.

The natural lake recorded lower average temperatures (25.8˚C) than the reservoir (31.9˚C) (Table 3) while both ranges were similar, suggesting both have surface water temperature driven by differences in elevation (Table 1). Average annual conductivity in the natural lake was ~7-fold higher (1991.4 μS/cm) than in the reservoir (290.6 μS/cm) increasing towards dry creek bed tributaries in the reservoir only (Figs 4 and 5) while dissolved oxygen was generally supersaturated in both systems.

^{222}Rn was ≤ 0.4 dpm/L^{-1} in the natural lake (Fig 4) and significantly higher in the reservoir during the drought period when it ranged from 0.6 dpm/L $^{-1}$ to 18.1 dpm/L $^{-1}$ (mean = 4.8 dpm/L^{-1}). ^{222}Rn decreased seasonally with increasing rainfall in the dry, wet transition and wet periods (Figs 5 and 6; Table 3).

CO_2 was undersaturated in the natural lake with the exception of a wet period (June, 2016) where ~20% of locations were supersaturated, reaching 451 μatm (Fig 4). The reservoir remained supersaturated in CO_2 throughout the year (Fig 5). The highest reservoir CO_2 ranges of 432 to 7647 μatm occurred in the drought period and were ~24-fold higher than that of the natural lake range (159–456 μatm) (Table 3). CO_2 followed the same spatial trend as ^{222}Rn concentrations increasing towards the reservoir dry creek bed tributaries although no flowing streams were visible (Fig 5A).

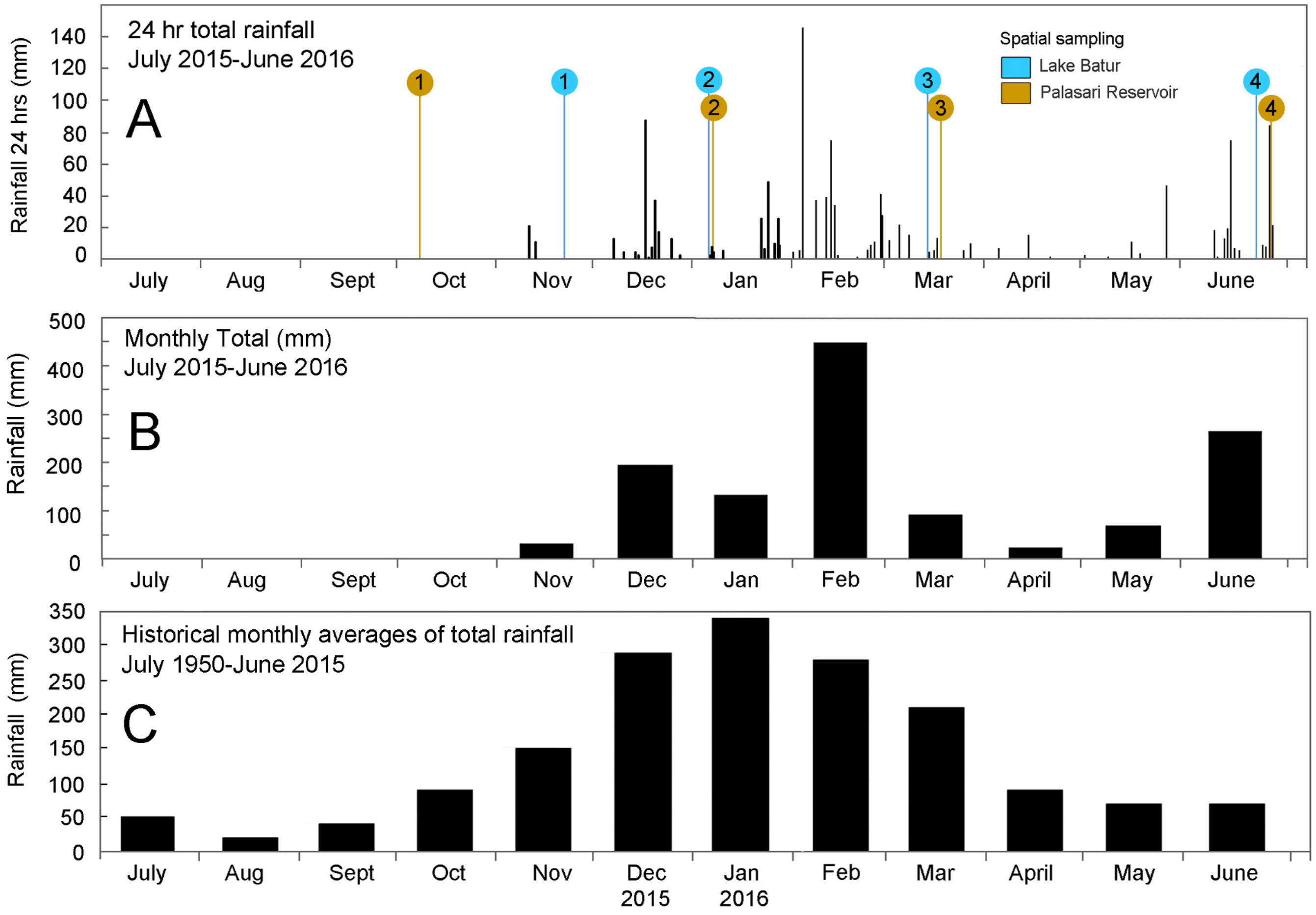

Fig 3. Rainfall time series compiled from raw rainfall datasets, indicating (A) underway seasonal sampling timeline for the natural lake, Lake Batur (blue) and the reservoir, Palasari Reservoir (brown); (B) rainfall monthly total from July 2015 to June 2016 and (C) historical monthly rainfall averages from 1950 to 2015 for Denpasar, Bali. Data accessed from NOAA, National Centre for Environmental Information (www.ncdc.noaa.gov/).

https://doi.org/10.1371/journal.pone.0198678.g003

Overall the lake was a sink of atmospheric CO_2 (average pCO_2 = 263.7±12.2 μatm) with average area weighted fluxes of -2.8±0.3 mmol m^{-2} d^{-1} tending towards atmospheric equilibrium in the wet period (average = 382.6±7.6 μatm) while the reservoir was a source with an average pCO_2 of 785.0±284 μatm and CO_2 evasion of 7.3±6.7 mmol m^{-2} d^{-1} (Figs 6 and 7; Table 4). CO_2 uptake increased in the natural lake from -2.4±0.4 mmol m^{-2} d^{-1} during the drought period to -4.5±0.3 mmol m^{-2} d^{-1} during the wet transition period, with a tendency towards atmospheric equilibrium in the wet period when the area-weighted flux was -0.3 mmol m^{-2} d^{-1}.

4. Discussion

4.1 Contrasting CO_2 dynamics in the natural lake and reservoir

We have assessed seasonal CO_2 dynamics on a natural and artificial lake in a tropical volcanic island, building on earlier work that focused mostly on boreal and temperate regions [37]. Volcanic caldera lakes such as Lake Batur typically have high groundwater recharge rates due to fracture-induced permeability [38], and small overall surface-groundwater interactions [39]. In contrast, artificial shallow lakes such as Palasari Reservoir typically have more pronounced

Table 3. Minimum, maximum, mean and standard deviations of underway variables (pCO_2, temperature, dissolved oxygen and conductivity) over 4 seasonal sampling periods in the natural lake (Lake Batur) (left) and the reservoir (Palasari Reservoir) (right).

		Lake Batur					Palasari Reservoir				
		**Drought**	**Dry**	**Wet transition**	**Wet**	Average	**Drought**	**Dry**	**Wet transition**	**Wet**	Average
pCO_2 (μatm)	Min	250.3	186.2	159.1	371.2	**241.7**	432.1	623.4	602.6	612.4	**567.6**
	Max	383.3	234.3	341.4	456.1	**353.8**	7647	832.7	989.6	1768.2	**2809.4**
	**Mean**	**285.5**	**202.5**	**184.3**	**382.6**	263.7	**676.3**	**696.2**	**725.8**	**1041.7**	785.0
	St. dev ±	18.7	10.8	11.5	7.6	**12.2**	659.3	42.7	92.9	339.5	**283.6**
^{222}Rn (dpm/L^{-1})	Min	0	0	0	0	**0.0**	0.6	0.2	0.1	0	**0.2**
	Max	0.3	0.2	0.3	0.4	**0.3**	18.1	4.6	3.2	2.8	**7.2**
	**Mean**	**0.1**	**0.01**	**0.04**	**0.09**	0.1	**4.8**	**2.5**	**0.9**	**0.8**	2.3
	St. Dev ±	0.1	0.04	0.07	0.14	**0.1**	5.3	1.4	0.9	0.9	**2.1**
Temp (°C)	Min	20.4	26.6	25.1	22.1	**23.6**	29.8	32.6	30.2	29.2	**30.5**
	Max	32	28.1	26.7	25.2	**28.0**	34.9	33.9	36.9	30.1	**34.0**
	**Mean**	**25.7**	**27.1**	**26**	**24.5**	25.8	**31.8**	**33.2**	**32.8**	**29.7**	31.9
	St. Dev ±	2.1	0.3	0.4	0.8	**0.9**	1.1	0.4	1.2	0.3	**0.8**
DO (%)	Min	61	77.4	81.3	56.9	**69.2**	77.3	74.3	53.5	85.7	**72.7**
	Max	194.9	244.5	213.9	93.4	**186.7**	339.7	432.9	328.9	121	**305.6**
	**Mean**	**124.1**	**144**	**136.5**	**86.7**	122.8	**151.9**	**141.4**	**135**	**105.8**	133.5
	St. Dev ±	32.6	31.9	30.1	4.8	**24.9**	54.7	50.7	59.7	11.1	**44.1**
Cond. (μS/cm^{-1})	Min	1385	1979	1952.3	2033	**1837.3**	305	321	220.0	184	**257.5**
	Max	1996	1984	2008.8	2289	**2069.5**	503	376	240.0	191	**327.5**
	**Mean**	**1989.5**	**1981.4**	**1973.9**	**2037**	1995.5	**321.9**	**333**	228.3	**187.8**	267.8
	St. Dev ±	104.6	6.3	7.4	1.6	**30.0**	34.6	10.4	3.3	2.4	**12.7**
Chl a (μg/L^{-1})	Min	0.1	2.48	2.3	3.3	**2.0**	0.4	1.3	2.4	No data	**1.4**
	Max	5.1	5.12	5.3	4.9	**5.1**	28.4	50.2	29.5	No data	**36.0**
	**Mean**	**1.96**	**3.43**	**3.9**	**4.3**	3.4	**4.61**	**6.98**	**6.2**	**No data**	5.9
	St. Dev ±	0.94	0.42	0.88	0.86	**0.8**	4.08	12.5	3.6	No data	**6.7**

https://doi.org/10.1371/journal.pone.0198678.t003

terrestrial sources that may stimulate productivity [40]. This is highlighted in the reservoir's elevated ranges of surface water pCO_2 with spatially variable measurements of surface water conductivity, dissolved oxygen and chlorophyll a (Figs 5 and 8; Table 3). This reflects seasonal rainfall influences with larger catchment sizes and watershed-to-reservoir ratios, when compared to the smaller catchments found in caldera lakes [41]. This is strongly shown in the differences in CO_2 concentrations within the natural lake and reservoir during the sampling period (Figs 4 and 5; Table 3). Although higher pCO_2 values were measured in the shallow (< 2m) near-shore zones in the reservoir, this did not result in large emissions when taking into account area weighted pCO_2 measurements (Fig 7).

For large and small systems such as Lake Batur and Palasari Reservoir, respectively, differences in CO_2 may be influenced by depth (due to the volume-to-sediment surface ratios), catchment area and lake area ratios (2:1 and 420:1 respectively; Table 1). These ratios exert strong influences over delivery of terrestrial organic matter and water chemistry [42]. For example, a study of 82 boreal lakes (areas = 0.04–1540 km2; max depth = 1–93 m) in Finland found that lake area and depth are important predictors of CO_2 evasion with higher emissions found in small, shallow lakes [43] In addition, nutrient delivery results from variations of surface water inflow dependent on regional rainfall regimes and geographic location. These traits

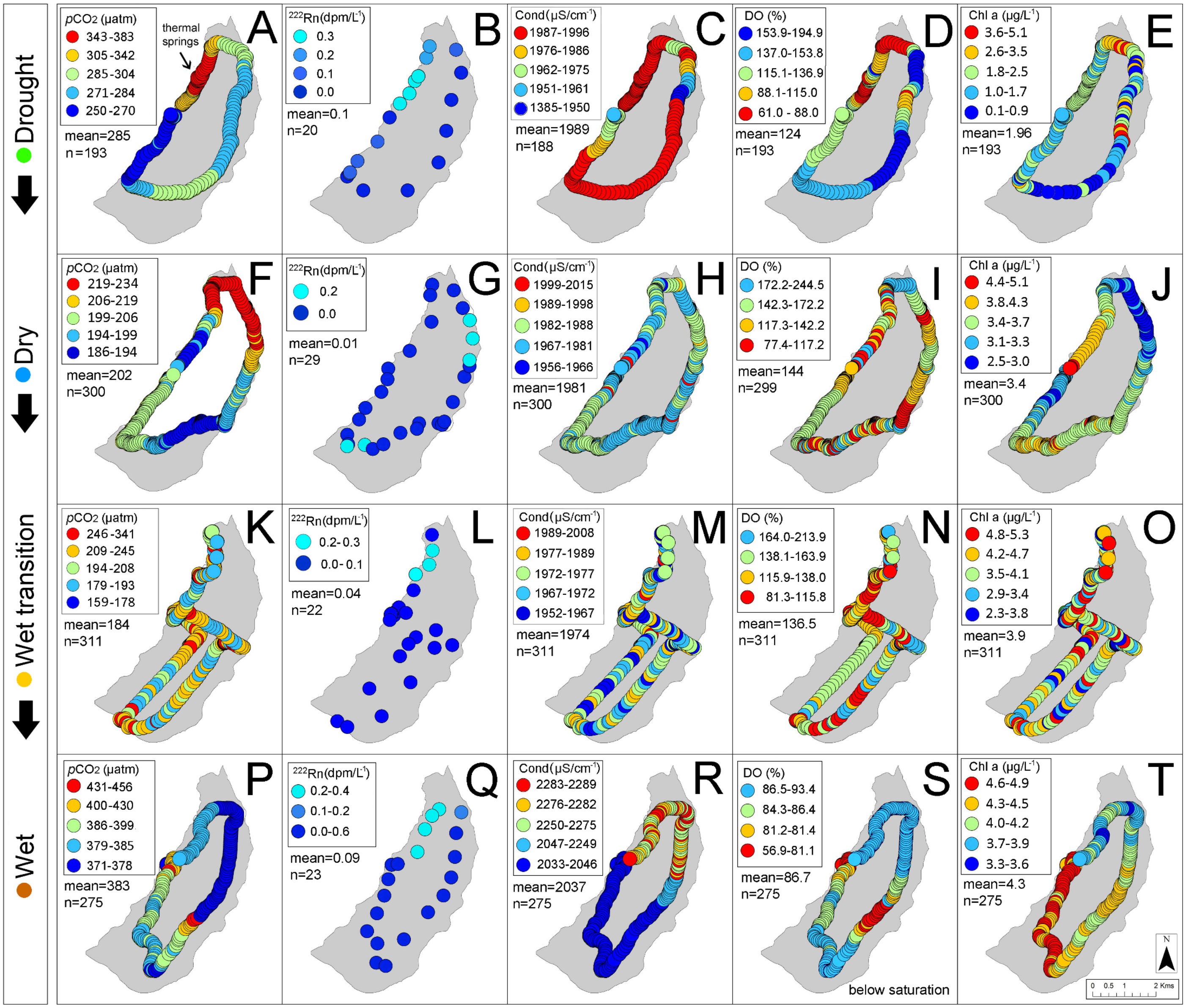

Fig 4. Seasonal underway measurements in Lake Batur showing the spatial distribution of *p*CO_2 and associated parameters. ^{222}Rn was measured in 1 minute intervals while *p*CO, conductivity, dissolved oxygen and chlorophyll a were measured in 10 minute intervals. Note the location of thermal springs (A) and different colour scale categories for each sampling period to highlight spatial patterns.

https://doi.org/10.1371/journal.pone.0198678.g004

are reflected in the lower average *p*CO_2 concentrations in the large, deep natural lake when compared to the small, shallow reservoir.

Correlations between CO_2 and conductivity, dissolved oxygen and chlorophyll a were similar in the deeper reservoir and in the natural lake (Figs 8 and 9). The strongest reservoir *p*CO_2 correlations with conductivity, DO and chlorophyll a were in the drought period ($r^2 = 0.77$; $p<0.0001$), wet period ($r^2 = 0.64$; $p<0.0001$) and wet transition period ($r^2 = 0.34$; $p<0.001$) respectively. However, the reservoir near-shore zone had more linear correlations typical of other lotic systems [44] but represented a minimal spatial area (see Fig 5A and Fig 8). In the natural lake, chlorophyll a accounted for 34% and 32% of the CO_2 variability in the dry and

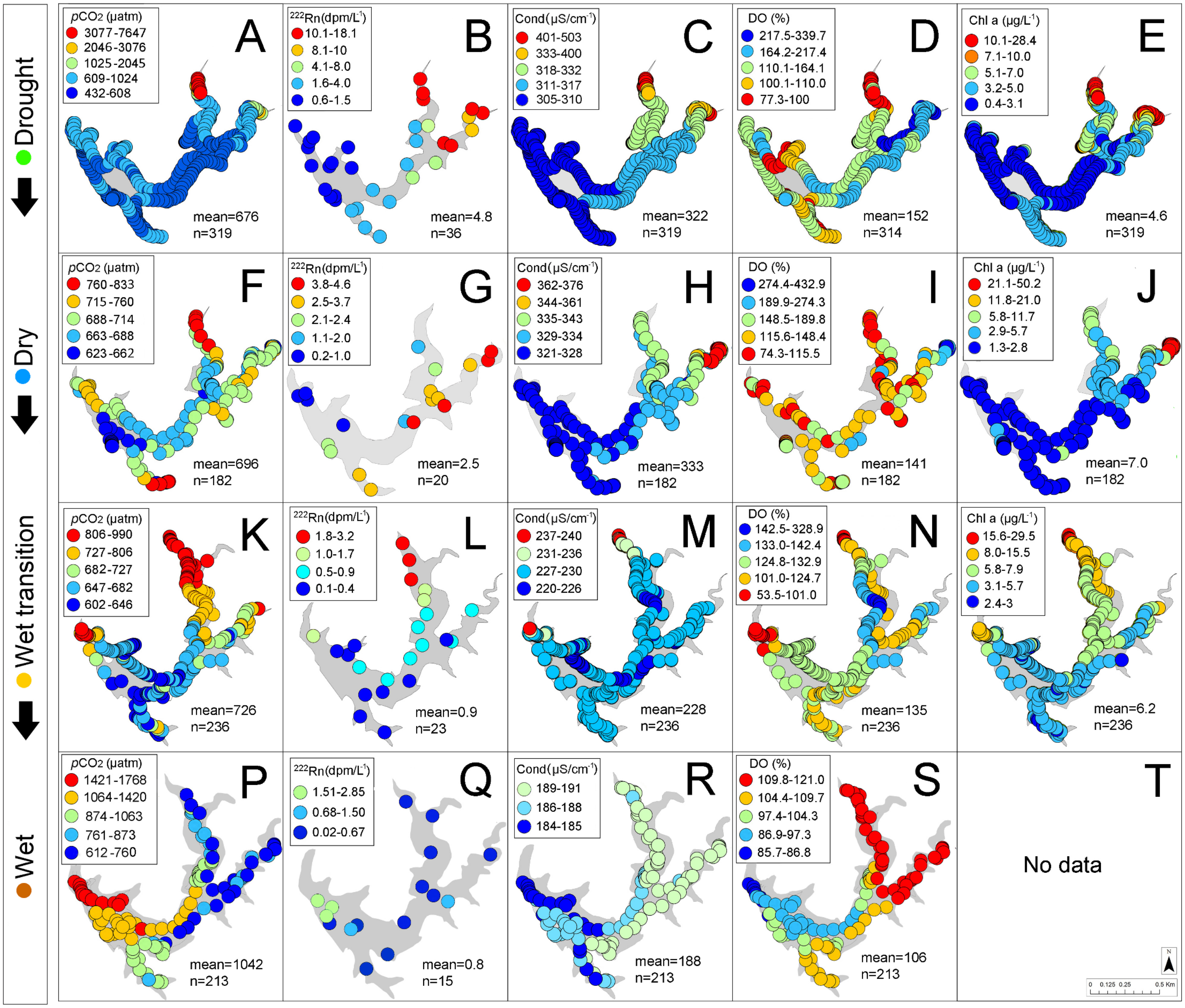

Fig 5. Seasonal underway measurements in Palasari Reservoir showing the spatial distribution of pCO_2 and associated parameters. ^{222}Rn was measured in 1 minute intervals while *p*CO, conductivity, dissolved oxygen and chlorophyll a were measured in 10 minute intervals. Note the location of a small stream (A) and different colour scale categories for each sampling period to highlight spatial patterns.

https://doi.org/10.1371/journal.pone.0198678.g005

wet period, respectively (Fig 9H and 9P). However, this relationship reversed from the drought to wet period. This reflected similarities with the reservoir which also showed a positive correlation between CO_2 and chlorophyll a (r^2 = 0.34) in the wet transition period (Fig 8L).

Several previous studies have used ^{222}Rn as a tracer in inland waters [28, 44, 45]. By simultaneously measuring the groundwater tracer ^{222}Rn and pCO_2, we found similarities to previous estuarine studies where radon followed CO_2 distributions [46, 47] (Figs 5 and 8). Groundwater inputs were apparently negligible in the natural lake (average ^{222}Rn 0.06 dpm/L^{-1}; Table 3) and was not considered as a significant driver of CO_2 dynamics. In the reservoir, ^{222}Rn decreased from the drought to wet periods. (Figs 5 and 8). Decreased wet period groundwater flow may

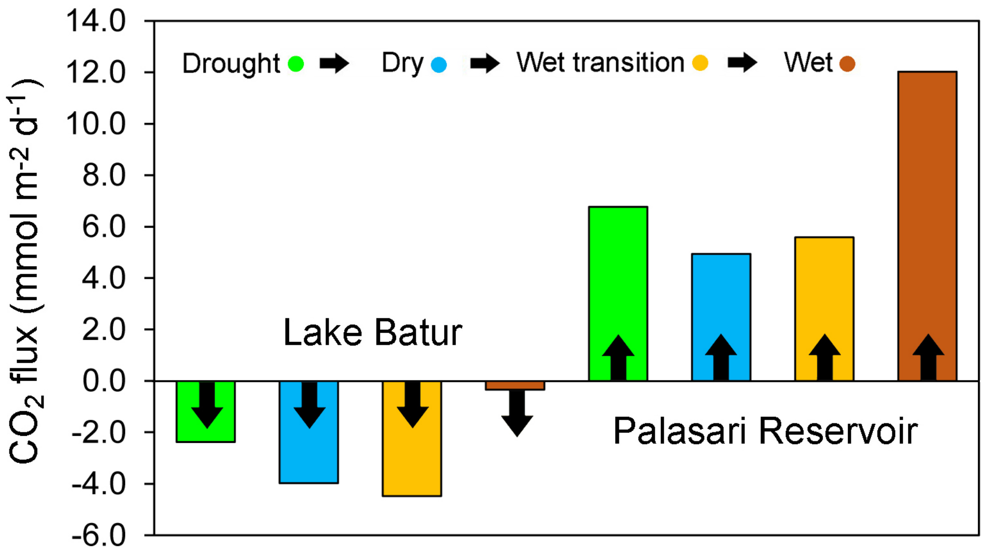

Fig 6. Average seasonal CO_2 fluxes (mmol m^{-2} d^{-1}) for Lake Batur (left) and Palasari Reservoir (right) with each survey period colour coded as drought (green), dry (blue), wet transition (orange) and wet (brown).

https://doi.org/10.1371/journal.pone.0198678.g006

be due to high surfacewater inputs with pCO_2 dynamics linked to this surfacewater loading [48]. Overall, our observations imply a stronger groundwater influence in the reservoir than in the natural lake.

4.2 Rainfall as a driver of CO_2 in tropical and temperate systems

Short, intense rainfall events, which are common in Bali's wet season, have recently been acknowledged as important pathways of terrestrial carbon loading to in inland waters [44, 48]. Artificial reservoirs and natural lakes receive CO_2 produced and derived from their catchment areas as a result of rainfall events when large amounts of carbon are rapidly transported to these waterbodies [49]. Many studies have reported correlations between atmospheric CO_2 fluxes and rainfall events [29, 50] with significant amounts of terrestrial CO_2 delivery to lake waters during these events [1].

In spite of the small sample size, we found a correlation between CO_2 and antecedent rainfall in the reservoir and lake (Fig 10). Rainfall events have previously been reported to deliver large amounts of particulate and dissolved organic carbon into aquatic systems [51, 52]. Higher seepage of CO_2 enriched groundwaters in the wet transition and wet sampling periods implies that wetter conditions lead to higher groundwater input due to a larger hydraulic head. In small lakes in northern Europe, CO_2 increased in the soil and lake following a significant rainfall event (61 mm). Terrestrial flushing was reflected in the high surface water CO_2 concentrations, with pCO_2 increasing from 1800 to 4370 μatm soon after the rain event [27].

In the reservoir, the effects of rainfall are emphasised by the decoupling of pCO_2 and ^{222}Rn which was elevated near a stream in the northern area (Fig 5). Tropical reservoir studies have reported rainfall events which load high amounts of terrestrial CO_2 into receiving waters [27, 29, 49] by increased river discharge [53] and carbon rich terrestrial inputs as a result of soil erosion [51]. Tropical regions in particular are prone to high CO_2 terrestrial loading as a result of episodic heavy rainfall events, pronounced wet seasons and high surface water temperatures. The low lying topography and plantations in the northern area of the reservoir may support more groundwater interactions (Fig 5A and Table 1). This is reflected in Fig 5 and

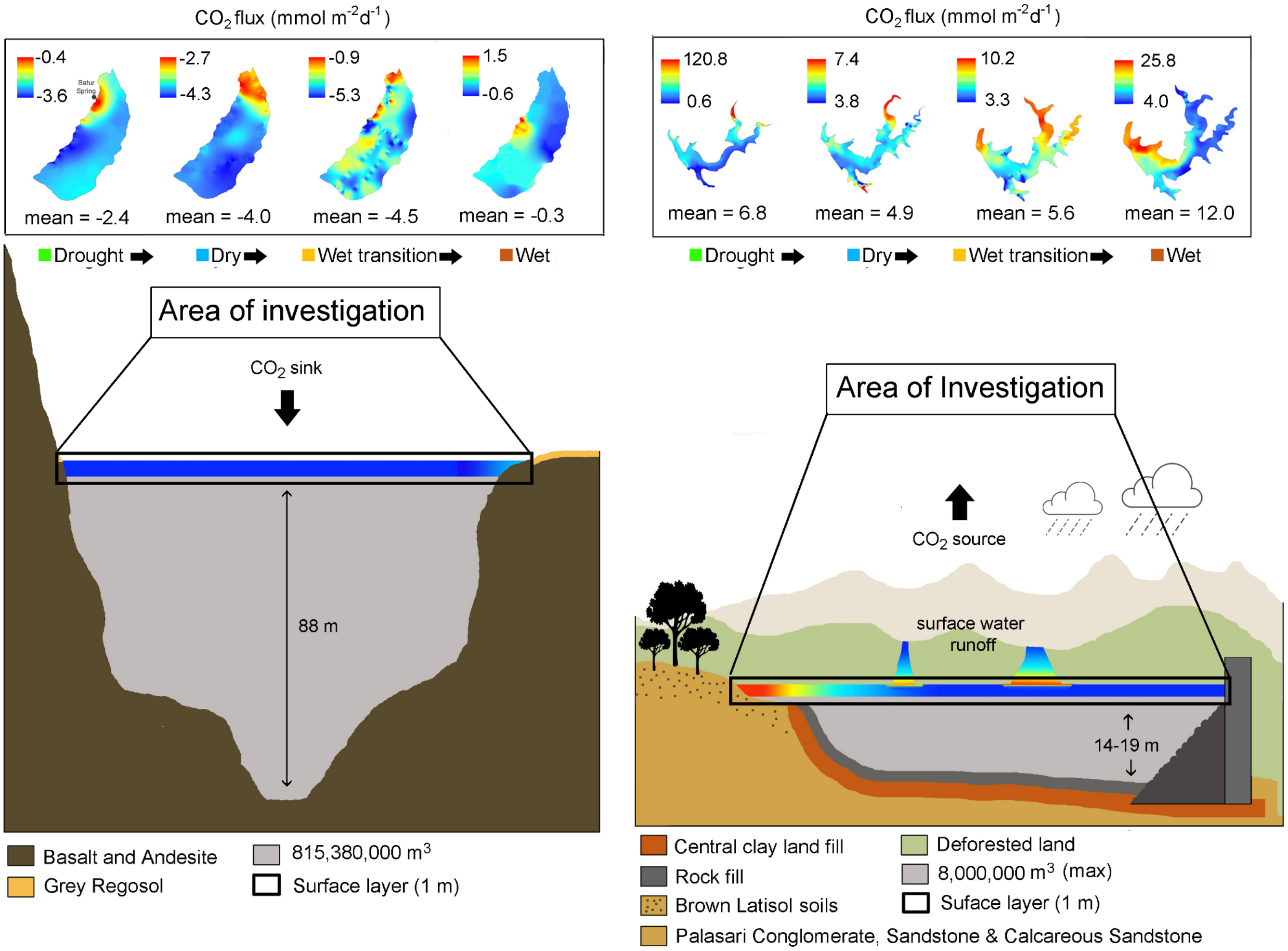

Fig 7. Area weighted seasonal CO_2 fluxes (mmol m^{-2} d^{-1}) averaged from the equations of six authors (Wanninkhof, 1992; MacIntyre et al., 1995; Cole & Carico, 1998; McGillis et al.,2001; Crusius and Wanninkhof, 2003; Cole et al.,2010) and interpolated using the ArcMap GIS spline with barriers method, through the drought, dry, wet transition and wet periods in Lake Batur (top) and Palasari Reservoir (bottom).

https://doi.org/10.1371/journal.pone.0198678.g007

supported by Fig 8, with outliers representing relatively small areas but reflecting the groundwater (^{222}Rn) dominated characteristics of the reservoir near-shore zone.

A recent study showed that water-to-air CO_2 fluxes in Brazilian lakes were significantly enhanced in heavy rainfall events, recording 28.5 ± 6.0 mmol CO_2 m^{-2} d^{-1} in dry periods and 245.3.1 ± 51.5 mmol CO_2 m^{-2} d^{-1} shortly after the heavy rainfall event. The increased inputs of CO_2 following periods of high rainfall were believed to be derived from increased inputs of CO_2 from groundwater to the lakes, resulting in an ~10-fold increase in lake pCO_2 [29]. Similarly, the natural lake from Bali sequestered atmospheric CO_2 throughout each sampling campaign but pCO_2 increased and approached atmospheric equilibrium in the wet period as a result of heavy rainfall events (Figs 10 and 11). The depth of the natural lake (mean = 50.8; max = 88 m; Table 1) may dilute elevations in pCO_2 as a result of terrestrial carbon inputs or groundwater inputs. Most previous lake investigations are from shallower systems [4, 37]. There is a paucity of data on CO_2 dynamics from such water bodies as the dominant caldera lakes in tropical volcanic regions such as Indonesia. Therefore, our observations may help to fill a gap in global CO_2 observations in lakes.

Table 4. Instantaneous windspeed and CO_2 fluxes of the 4 different transfer velocity parameterizations (see Table 2) for the natural lake (Lake Batur) and the reservoir (Palasari Reservoir) throughout the sampling period. Area weighted CO_2 fluxes for Lake Batur (left) and Palasari Reservoir (right) and interpolated GPS point data using the Arcmap GIS spline with barriers interpolation method.

		Lake Batur						Palasarai Reservoir				
**Code**		**Drought**	**Dry**	**Wet transition**	**Wet**	**Average**		**Drought**	**Dry**	**Wet transition**	**Wet**	**Average**
		CO_2 fluxes (mmol m^{-2} d^{-1})						*CO_2 fluxes (mmol m^{-2} d^{-1})*				
W92	Min	-4.1	-5.7	-7	-0.8	**-4.4**		0.9	6.3	5.6	5.8	**4.7**
	Max	-0.5	-3.7	-1.2	1.8	**-0.9**		212.6	12.3	16.6	37.1	**69.7**
	Mean	-3.1	-5.3	-5.8	-0.4	**-3.7**		11.4	8.2	9.3	17.4	**11.6**
	St Dev ±	0.5	0.3	0.3	0.2	**0.3**		32.2	1	2.7	9.2	**11.3**
M95	Min	-3.6	-5.1	-6.4	-0.7	**-4.0**		0.8	5.6	5	5.1	**4.1**
	Max	-0.4	-3.3	-0.9	1.7	**-0.7**		189.5	11	14.8	33.1	**62.1**
	Mean	-2.7	-4.7	-5.1	-0.4	**-3.2**		10.1	7.3	8.2	15.5	**10.3**
	St Dev ±	0.5	0.3	0.3	0.2	**0.3**		28.7	0.9	1.9	8.2	**9.9**
C&C98	Min	-4.5	-4.8	-6	-0.7	**-4.0**		0.6	3.5	2.9	4.1	**2.8**
	Max	-0.4	-3.1	-1	1.7	**-0.7**		103.9	6.7	9.6	27.3	**36.9**
	Mean	-2.7	-4.4	-5.1	-0.3	**-3.1**		6.2	4.6	5.2	12.6	**7.2**
	St Dev ±	0.4	0.3	0.3	0.2	**0.3**		15.6	0.4	1.4	6.8	**6.1**
M01	Min	-5.2	-5.5	-6.9	-0.9	**-4.6**		0.7	4	3.4	4.8	**3.2**
	Max	-0.5	-3.6	-1.2	2.1	**-0.8**		120.1	7.8	11	31.6	**42.6**
	Mean	-3.1	-5.1	-5.9	-0.5	**-3.7**		7.1	5.3	5.9	14.6	**8.2**
	St Dev ±	0.5	0.3	0.4	0.3	**0.4**		18	0.5	1.6	7.9	**7.0**
C&W03	Min	-3.5	-3.8	-4.8	-0.6	**-3.2**		0.5	2.8	2.3	3.3	**2.2**
	Max	-0.3	-2.4	-0.8	1.4	**-0.5**		82.2	5.3	7.6	21.6	**29.2**
	Mean	-2.3	-3.5	-4.1	-0.3	**-2.6**		4.9	3.6	4.1	10	**5.7**
	St Dev ±	0.4	0.2	0.2	0.2	**0.3**		12.3	0.4	1.1	5.4	**4.8**
C10	Min	-0.7	-0.8	-1	-0.1	**-0.7**		0.1	0.5	0.5	0.6	**0.4**
	Max	-0.1	-0.5	-0.2	0.3	**-0.1**		16.3	1.1	1.5	4.3	**5.8**
	Mean	-0.4	-0.7	-0.8	-0.1	**-0.5**		1	0.7	0.8	2	**1.1**
	St Dev ±	0.1	0.04	0.1	0.04	**0.1**		2.4	0.1	0.2	1.1	**1.0**
**Average**	Min	-3.6	-4.3	-5.3	-0.6	**-3.5**		0.6	3.8	3.3	4	**2.9**
**Combined**	Max	-0.4	-2.7	-0.9	1.5	**-0.6**		120.8	7.4	10.2	25.8	**41.1**
**Flux**	**Mean**	**-2.4**	**-4**	**-4.5**	**-0.3**	-2.8		**6.8**	**4.9**	**5.6**	**12**	7.3
	St Dev ±	0.4	0.2	0.3	0.2	**0.3**		18.2	0.5	1.5	6.4	**6.7**

https://doi.org/10.1371/journal.pone.0198678.t004

4.3 Implications

Due to uneven spatial and temporal distribution of rainfall and river flow, reservoir construction is becoming increasingly important in regions of fast population growth such as Southeast Asia. We speculate that the accelerated construction of reservoirs [54, 55] and population growth [55] will increase the contribution of Southeast Asia inland waters to the global carbon budget. In Bali, the current reservoir capacity has a ratio of storage per capita of only 63.5 m^3 which is inadequate to cope with the increasing water demand (Direktorat Jenderal Sumber Daya Air: http://sda.pu.go.id/). Bali's local population of 4,200,000 in 2012 had a water demand of 229,950,000 m^3 $yr^{-1.}$ This does not take into account irrigation water for rice, agriculture, industry and tourism growing 20% between 2015 and 2016. Assuming future reservoir construction will supply water demand, reservoirs may become a more significant regional CO_2 source that will need to be managed effectively. In 2015, Indonesia had 6 reservoirs under

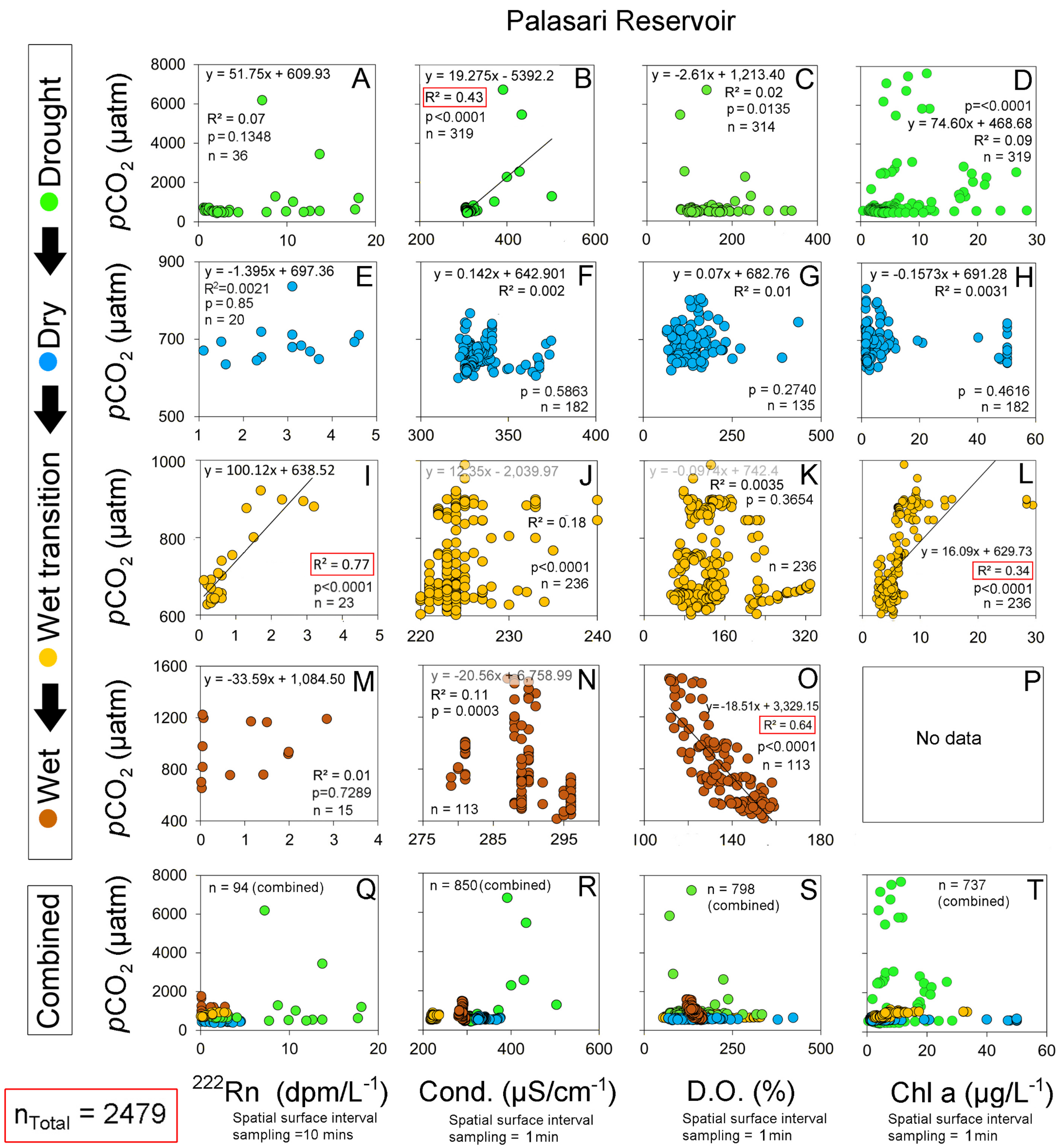

Fig 8. Relationships between pCO_2 and ^{222}Rn (groundwater tracer), conductivity, dissolved oxygen and chlorophyll a (from left to right) in reservoir (Palasari Reservoir).

https://doi.org/10.1371/journal.pone.0198678.g008

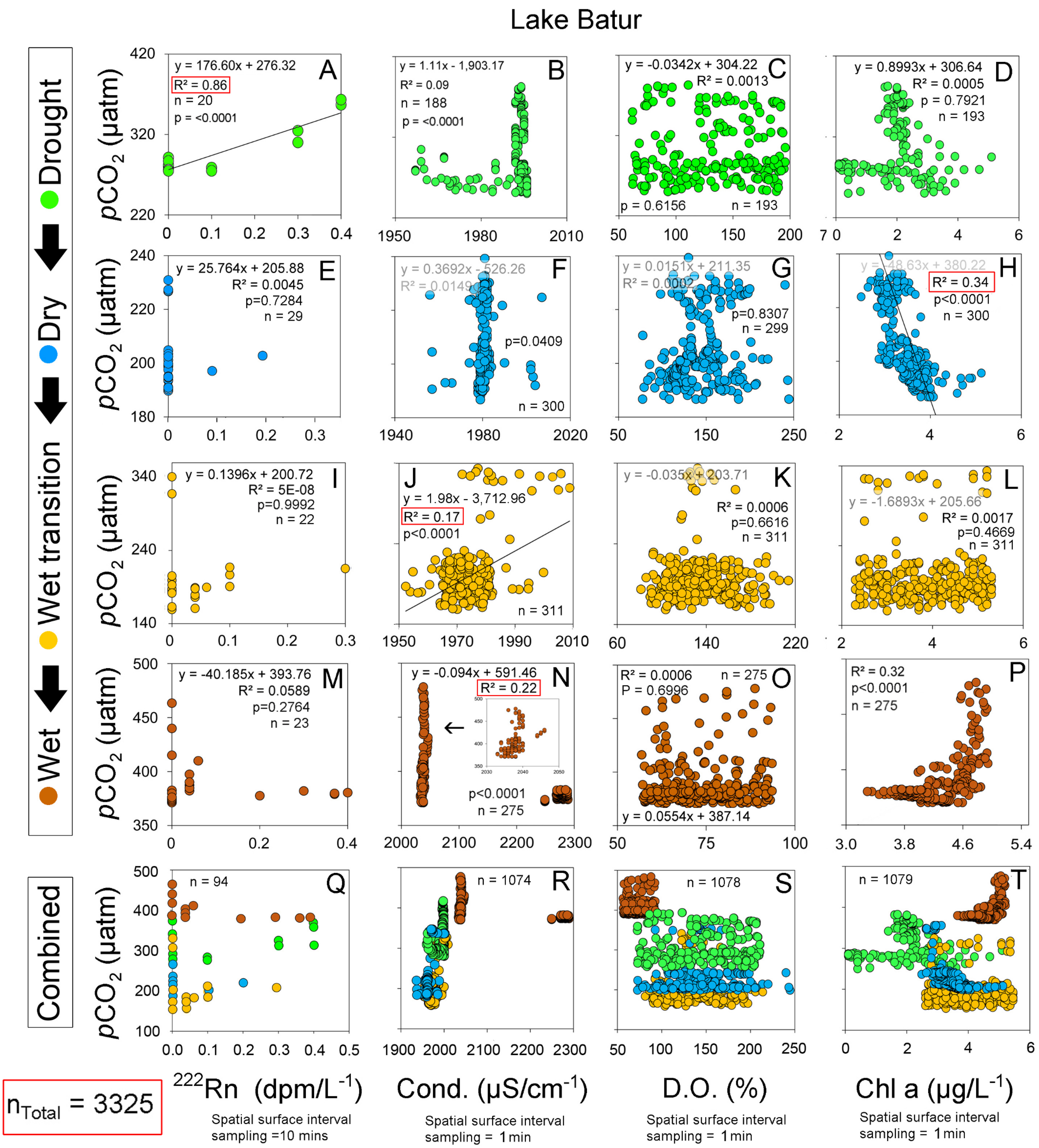

Fig 9. Relationships between pCO_2 and ^{222}Rn (groundwater tracer), conductivity, dissolved oxygen and chlorophyll a (from left to right) in the natural lake (Lake Batur).

https://doi.org/10.1371/journal.pone.0198678.g009

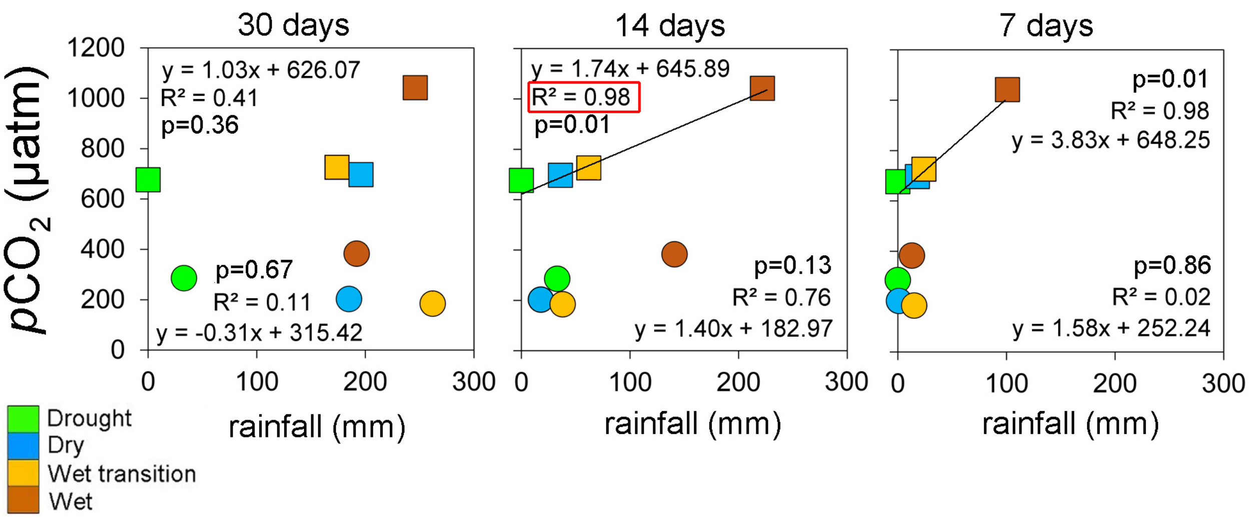

Fig 10. Rainfall (30, 14 and 7 days prior) and *p*CO_2 dynamics in Lake Batur (circles) and Palasari Reservoir (squares) indicating differences in average *p*CO_2 in the confined and open lake in drier periods and elevated average *p*CO_2 in both systems after heavy rainfall.

https://doi.org/10.1371/journal.pone.0198678.g010

construction, 6 in negotiation stages and 7 in design stages, not taking into account current small reservoir construction (www.narbo.jp/).

Lakes and reservoirs in tropical regions, as a result of stable temperature and light, have been recently reported to have lower seasonal variations of biological activity when compared to boreal and temperate counterparts [56]. For example low variations of seasonal CO_2 concentrations in a tropical lake (Lake Kivu, East Africa) were linked to tropical climate and partly associated with minimal water temperature variations [56]. Predicted increases in both tropical monsoonal temperatures and reservoir construction may increase the inland water contribution to the global carbon budget.

Tropical lakes and reservoirs are under-represented and comprise only 1.5% of the global dataset (n = 7939) of CO_2 emissions (Raymond et al., 2013). While tropical lakes may be responsible for 34% of the global atmospheric CO_2 fluxes from inland waters, they cover only 2.4% of the global lake area [1]. Lake ranges and averages of *p*CO_2 were found to be amongst the highest in tropical regions. Rainfall and temperature appeared to be a strong controls over *p*CO_2 in our study. The predicted temperature increase would increase bacterial metabolism resulting in more organic carbon respiration. Due to poor representation in global datasets, constraining CO_2 fluxes in tropical lakes and reservoirs is particularly important [4].

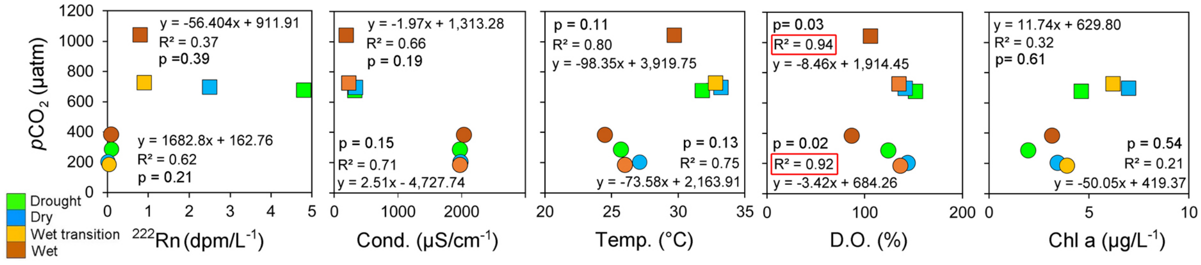

Fig 11. Seasonal averages for the drought, wet, wet transition and wet periods for Lake Batur (circle) and Palasari Reservoir (square), indicating relationships between CO_2 and ancillary variables.

https://doi.org/10.1371/journal.pone.0198678.g011

5. Conclusions

Our observations in Bali revealed that antecedent rainfall seems to be a major control on seasonal CO_2 distributions in both the lake and reservoir. The spatial distribution of pCO_2 was driven primarily by autochthonous processes (water column metabolism) in the deep lake, and allochthonous processes (groundwater seepage) in the shallow reservoir. Overall, the natural lake was an atmospheric CO_2 sink, while the reservoir was releasing CO_2 to the atmosphere. We speculate that the predicted increase in reservoir area in tropical regions may increase CO_2 fluxes to the atmosphere and partially offset the sink provided by deep, volcanic, natural lakes. Site specific carbon investigations are needed to monitor inland waters on a regional scale. Due to the rapid expansion of reservoir construction, particularly in tropical regions, it may be necessary to develop long term monitoring programs that capture reservoir evolution and infilling process as well as large scale comparative studies already available for better studied northern hemisphere lakes.

Acknowledgments

Paul Anthony Macklin was supported by the Australian Government Research Training Program Scholarship. We acknowledge Ceylena Holloway from the National Marine Science Centre for support with research instrumentation. The Ministry of Research, Technology and Higher Education of the Republic of Indonesia (RISTEKDIKTI); The Ministry of Home Affairs of the Republic of Indonesia (MoHA); the Direktorat Jenderal Sumber Daya Air and Governor of Bali I Made Mangku Pastika are acknowledged for access and permissions to sample to inland water sites. We acknowledge support from the Australian Research Council (DE140101733, DE150100581, and LE120100156) that partially funded IRS and DTM salaries and the portable analytical instrumentation.

Author Contributions

Conceptualization: Paul A. Macklin, Damien T. Maher, Isaac R. Santos.

Data curation: Paul A. Macklin, I. Gusti Ngurah Agung Suryaputra.

Formal analysis: Paul A. Macklin, I. Gusti Ngurah Agung Suryaputra, Isaac R. Santos.

Investigation: Paul A. Macklin, I. Gusti Ngurah Agung Suryaputra.

Methodology: Paul A. Macklin, Damien T. Maher.

Project administration: Paul A. Macklin, I. Gusti Ngurah Agung Suryaputra, Isaac R. Santos.

Resources: Paul A. Macklin, I. Gusti Ngurah Agung Suryaputra, Damien T. Maher.

Supervision: Damien T. Maher, Isaac R. Santos.

Validation: Paul A. Macklin, I. Gusti Ngurah Agung Suryaputra, Isaac R. Santos.

Visualization: Paul A. Macklin.

Writing – original draft: Paul A. Macklin.

Writing – review & editing: I. Gusti Ngurah Agung Suryaputra, Damien T. Maher, Isaac R. Santos.

References

1. Raymond PA, Hartmann J, Lauerwald R, Sobek S, McDonald C, Hoover M, et al. Global carbon dioxide emissions from inland waters. Nature. 2013; 503(7476):355–9. https://doi.org/10.1038/nature12760 PMID: 24256802

2. Battin TJ, Kaplan LA, Findlay S, Hopkinson CS, Marti E, Packman AI, et al. Biophysical controls on organic carbon fluxes in fluvial networks. Nature Geoscience. 2008; 1(2):95–100.
3. Dean WE, Gorham E. Magnitude and significance of carbon burial in lakes, reservoirs, and peatlands. Geology. 1998; 26(6):535–8.
4. Tranvik LJ, Downing JA, Cotner JB, Loiselle SA, Striegl RG, Ballatore TJ, et al. Lakes and reservoirs as regulators of carbon cycling and climate. Limnol Oceanogr. 2009; 54(6:2):2298–314.
5. Postel SL. Entering an era of water scarcity: the challenges ahead. Ecol Appl. 2000; 10(4):941–8.
6. Louis VLS, Kelly CA, Duchemin É, Rudd JW, Rosenberg DM. Reservoir Surfaces as Sources of Greenhouse Gases to the Atmosphere: A Global Estimate. Bioscience. 2000; 50(9):766–75.
7. Downing JA, Prairie Y, Cole J, Duarte C, Tranvik L, Striegl R, et al. The global abundance and size distribution of lakes, ponds, and impoundments. Limnol Oceanogr. 2006; 51(5):2388–97.
8. Syvitski JP, Vörösmarty CJ, Kettner AJ, Green P. Impact of humans on the flux of terrestrial sediment to the global coastal ocean. Science. 2005; 308(5720):376–80. https://doi.org/10.1126/science.1109454 PMID: 15831750
9. Lapierre JF, Seekell DA, Giorgio PA. Climate and landscape influence on indicators of lake carbon cycling through spatial patterns in dissolved organic carbon. Glob Chang Biol. 2015; 21(12):4425–35. https://doi.org/10.1111/gcb.13031 PMID: 26150108
10. Li S, Bush RT, Mao R, Xiong L, Ye C. Extreme drought causes distinct water acidification and eutrophication in the Lower Lakes (Lakes Alexandrina and Albert), Australia. J Hydrol (Amst). 2017; 544:133–46.
11. Humborg C, Mörth CM, Sundbom M, Borg H, Blenckner T, Giesler R, et al. CO_2 supersaturation along the aquatic conduit in Swedish watersheds as constrained by terrestrial respiration, aquatic respiration and weathering. Glob Chang Biol. 2010; 16(7):1966–78.
12. Marcé R, Obrador B, Morguí J-A, Riera JL, López P, Armengol J. Carbonate weathering as a driver of CO_2 supersaturation in lakes. Nat Geosci. 2015; 8(2):107.
13. Cole JJ, Prairie YT, Caraco NF, McDowell WH, Tranvik LJ, Striegl RG, et al. Plumbing the global carbon cycle: integrating inland waters into the terrestrial carbon budget. Ecosystems. 2007; 10(1):172–85.
14. Panneer Selvam B, Natchimuthu S, Arunachalam L, Bastviken D. Methane and carbon dioxide emissions from inland waters in India–implications for large scale greenhouse gas balances. Global Change Biol. 2014; 20(11):3397–407.
15. Gerardo-Nieto O, Astorga-España MS, Mansilla A, Thalasso F. Initial report on methane and carbon dioxide emission dynamics from sub-Antarctic freshwater ecosystems: A seasonal study of a lake and a reservoir. Sci Total Environ. 2017; 593:144–54. https://doi.org/10.1016/j.scitotenv.2017.02.144 PMID: 28342415
16. Guérin F, Abril G, Serça D, Delon C, Richard S, Delmas R, et al. Gas transfer velocities of CO_2 and CH_4 in a tropical reservoir and its river downstream. J Mar Syst. 2007; 66(1–4):161–72.
17. McGowan S, Anderson NJ, Edwards ME, Langdon PG, Jones VJ, Turner S, et al. Long-term perspectives on terrestrial and aquatic carbon cycling from palaeolimnology. WIREs Water. 2016; 3(2):211–34.
18. Abril G, Etcheber H, Borges AV, Frankignoulle M. Excess atmospheric carbon dioxide transported by rivers into the Scheldt estuary. Comptes Rendus de l'Academie des Sciences-Series IIA-Earth and Planetary Science. 2000; 330(11):761–8.
19. Kessler TJ, Harvey CF. The global flux of carbon dioxide into groundwater. Geophys Res Lett. 2001; 28 (2):279–82.
20. Hagedorn B, Cartwright I. The CO_2 system in rivers of the Australian Victorian Alps: CO_2 evasion in relation to system metabolism and rock weathering on multi-annual time scales. Appl Geochem. 2010; 25 (6):881–99.
21. Raymond PA, Bauer JE, Cole JJ. Atmospheric CO_2 evasion, dissolved inorganic carbon production, and net heterotrophy in the York River estuary. Limnol Oceanogr. 2000; 45(8):1707–17.
22. Vachon D, Del Giorgio PA. Whole-lake CO_2 dynamics in response to storm events in two morphologically different lakes. Ecosystems. 2014; 17(8):1338–53.
23. Macklin PA, Maher DT, Santos IR. Estuarine canal estate waters: Hotspots of CO_2 outgassing driven by enhanced groundwater discharge? Marine Chemistry. 2014; 167:82–92.
24. Sulman B. Impact of hydrological variations on modeling of peatland CO_2 fluxes: Results from the North American Carbon Program site synthesis. J Geophys Res. 2012; 117:G01031.
25. Sadat-Noori M, Maher DT, Santos IR. Groundwater discharge as a source of dissolved carbon and greenhouse gases in a subtropical estuary. Estuaries and Coasts. 2016; 39(3):639–56.

26. Santos IR, Maher DT, Eyre BD. Coupling automated radon and carbon dioxide measurements in coastal waters. Environ Sci Technol. 2012; 46(14):7685–91. https://doi.org/10.1021/es301961b PMID: 22694256

27. Rasilo T, Ojala A, Huotari J, Pumpanen J. Rain induced changes in carbon dioxide concentrations in the soil–lake–brook continuum of a boreal forested catchment. Vadose Zone Journal. 2012; 11(2).

28. Perkins AK, Santos IR, Sadat-Noori M, Gatland JR, Maher DT. Groundwater seepage as a driver of CO_2 evasion in a coastal lake (Lake Ainsworth, NSW, Australia). Environmental Earth Sciences. 2015; 74(1):779–92.

29. Marotta H, Duarte C, Pinho L, Enrich-Prast A. Rainfall leads to increased pCO_2 in Brazilian coastal lakes. Biogeosciences. 2010; 7(5):1607–14.

30. Moosdorf N, Stieglitz T, Waska H, Dürr HH, Hartmann J. Submarine groundwater discharge from tropical islands: a review. Grundwasser. 2015; 20(1):53–67.

31. Sulastri M. Inland water resources and limnology in Indonesia. Tropics. 2006; 15(3):285–95.

32. Dulaiova H, Peterson R, Burnett W, Lane-Smith D. A multi-detector continuous monitor for assessment of ^{222}Rn in the coastal ocean. J Radioanal Nucl Chem. 2005; 263(2):361–3.

33. Weiss RF. Carbon dioxide in water and seawater: the solubility of a non-ideal gas. Mar Chem. 1974; 2 (3):203–15.

34. Schubert M, Paschke A, Lieberman E, Burnett WC. Air–water partitioning of ^{222}Rn and its dependence on water temperature and salinity. Environ Sci Technol. 2012; 46(7):3905–11. https://doi.org/10.1021/es204680n PMID: 22385122

35. Wanninkhof R. Relationship between wind speed and gas exchange over the ocean. J Geophys Res Oceans. 1992; 97(C5):7373–82.

36. Maher DT, Eyre BD. Carbon budgets for three autotrophic Australian estuaries: Implications for global estimates of the coastal air-water CO_2 flux. Global Biogeochem Cycles. 2012;26(1).

37. Sobek S, Tranvik LJ, Cole JJ. Temperature independence of carbon dioxide supersaturation in global lakes. Global Biogeochem Cycles. 2005; 19(2).

38. Sanford WE, Konikow LF, Rowe Jr GL, Brantley SL. Groundwater transport of crater-lake brine at Poa´ s Volcano, Costa Rica. J Volcanol Geotherm Res. 1995; 64(3–4):269–93.

39. Varekamp J, Kreulen R. The stable isotope geochemistry of volcanic lakes, with examples from Indonesia. J Volcanol Geotherm Res. 2000; 97(1–4):309–27.

40. Williamson CE, Saros JE, Vincent WF, Smol JP. Lakes and reservoirs as sentinels, integrators, and regulators of climate change. Limnol Oceanogr. 2009; 54:2273–82.

41. Knoll LB, Vanni MJ, Renwick WH, Dittman EK, Gephart JA. Temperate reservoirs are large carbon sinks and small CO_2 sources: Results from high-resolution carbon budgets. Global Biogeochem Cycles. 2013; 27(1):52–64.

42. Sobek S, Tranvik LJ, Prairie YT, Kortelainen P, Cole JJ. Patterns and regulation of dissolved organic carbon: An analysis of 7,500 widely distributed lakes. Limnol Oceanogr. 2007; 52(3):1208–19.

43. Kortelainen P, Rantakari M, Pajunen H, Huttunen JT, Mattsson T, Juutinen S, et al. Carbon evasion/accumulation ratio in boreal lakes is linked to nitrogen. Global Biogeochem Cycles. 2013; 27(2):363–74.

44. Looman A, Santos IR, Tait DR, Webb JR, Sullivan CA, Maher DT. Carbon cycling and exports over diel and flood-recovery timescales in a subtropical rainforest headwater stream. Sci Total Environ. 2016; 550:645–57. https://doi.org/10.1016/j.scitotenv.2016.01.082 PMID: 26849329

45. Dimova NT, Burnett WC, Chanton JP, Corbett JE. Application of radon-222 to investigate groundwater discharge into small shallow lakes. J Hydrol (Amst). 2013; 486:112–22.

46. Maher DT, Cowley K, Santos IR, Macklin P, Eyre BD. Methane and carbon dioxide dynamics in a subtropical estuary over a diel cycle: Insights from automated in situ radioactive and stable isotope measurements. Mar Chem. 2015; 168:69–79.

47. Santos IR, Beck M, Brumsack H-J, Maher DT, Dittmar T, Waska H, et al. Porewater exchange as a driver of carbon dynamics across a terrestrial-marine transect: Insights from coupled ^{222}Rn and pCO_2 observations in the German Wadden Sea. Mar Chem. 2015; 171:10–20.

48. Webb JR, Santos IR, Robson B, Macdonald B, Jeffrey L, Maher DT. Constraining the annual groundwater contribution to the water balance of an agricultural floodplain using radon: The importance of floods. Water Resour Res. 2017; 53(1):544–62.

49. Vähätalo AV, Salonen K, Münster U, Järvinen M, Wetzel RG. Photochemical transformation of allochthonous organic matter provides bioavailable nutrients in a humic lake. Archiv für Hydrobiologie. 2003; 156(3):287–314.

50. Rantakari M, Kortelainen P. Interannual variation and climatic regulation of the CO_2 emission from large boreal lakes. Global Change Biol 2005; 11(8):1368–80.

51. Van Oost K, Quine T, Govers G, De Gryze S, Six J, Harden J, et al. The impact of agricultural soil erosion on the global carbon cycle. Science. 2007; 318(5850):626–9. https://doi.org/10.1126/science.1145724 PMID: 17962559

52. Atkins ML, Santos IR, Maher DT. Seasonal exports and drivers of dissolved inorganic and organic carbon, carbon dioxide, methane and $\delta^{13}C$ signatures in a subtropical river network. Sci Total Environ. 2017; 575:545–63. https://doi.org/10.1016/j.scitotenv.2016.09.020 PMID: 27692471

53. Prasad M, Sarma V, Sarma V, Krishna M, Reddy N. Carbon Dioxide Emissions from the Tropical Dowleiswaram Reservoir on the Godavari River, Southeast of India. J Water Resource Prot. 2013; 5 (05):534.

54. Li S, Gu S, Tan X, Zhang Q. Water quality in the upper Han River basin, China: the impacts of land use/land cover in riparian buffer zone. J Hazard Mater. 2009; 165(1):317–24.

55. Lapierre JF, Seekell DA, Giorgio PA. Climate and landscape influence on indicators of lake carbon cycling through spatial patterns in dissolved organic carbon. Global Change Biol. 2015; 21(12):4425–35.

56. Borges A, Morana C, Bouillon S, Servais P, Descy J, Darchambeau F. Carbon cycling of Lake Kivu (East Africa): net autotrophy in the epilimnion and emission of CO_2 to the atmosphere sustained by geogenic inputs. PloS one. 2014; 9(10):e109500. https://doi.org/10.1371/journal.pone.0109500 PMID: 25314144

57. MacIntyre S, Wanninkhof R, Chanton J. Trace gas exchange across the air-water interface in freshwater and coastal marine environments. Biogenic trace gases: Measuring emissions from soil and water. 1995; 5297.

58. Cole JJ, Caraco NF. Atmospheric exchange of carbon dioxide in a low-wind oligotrophic lake measured by. Limnol Oceanogr. 1998; 43(4):647–56.

59. McGillis WR, Edson J, Hare J, Fairall C. Direct covariance air-sea CO_2 fluxes. J Geophys Res Oceans. 2001; 106(C8):16729–45.

60. Crusius J, Wanninkhof R. Gas transfer velocities measured at low wind speed over a lake. Limnol Oceanogr. 2003; 48(3):1010–7.

61. Cole JJ, Bade DL, Bastviken D, Pace ML, Van de Bogert M. Multiple approaches to estimating air-water gas exchange in small lakes. Limnol Oceanogr Methods. 2010; 8(6):285–93.
